# Interactions of the *Bacillus subtilis* DnaE polymerase with replisomal proteins modulate its activity and fidelity

**DOI:** 10.1098/rsob.170146

**Published:** 2017-09-06

**Authors:** Vasileios Paschalis, Emmanuelle Le Chatelier, Matthew Green, François Képès, Panos Soultanas, Laurent Janniere

**Affiliations:** 1 Centre for Biomolecular Sciences, School of Chemistry, University of Nottingham, University Park, Nottingham NG7 2RD, UK; 2 Institut National de la Recherche Agronomique, Génétique Microbienne, 78350 Jouy-en-Josas, France; 3 iSSB, Genopole, CNRS, Univ EVRY, Université Paris-Saclay, Génopole Campus 1, Genavenir 6, 5 rue Henri Desbruères, 91030 Evry, France

**Keywords:** DNA replication, DNA polymerase, single-strand binding protein, DNA polymerase clamp, DNA polymerase proofreading, mismatch repair

## Abstract

During *Bacillus subtilis* replication two replicative polymerases function at the replisome to collectively carry out genome replication. In a reconstituted *in vitro* replication assay, PolC is the main polymerase while the lagging strand DnaE polymerase briefly extends RNA primers synthesized by the primase DnaG prior to handing-off DNA synthesis to PolC. Here, we show *in vivo* that (i) the polymerase activity of DnaE is essential for both the initiation and elongation stages of DNA replication, (ii) its error rate varies inversely with PolC concentration, and (iii) its misincorporations are corrected by the mismatch repair system post-replication. We also found that the error rates in cells encoding mutator forms of both PolC and DnaE are significantly higher (up to 15-fold) than in PolC mutants. *In vitro*, we showed that (i) the polymerase activity of DnaE is considerably stimulated by DnaN, SSB and PolC, (ii) its error-prone activity is strongly inhibited by DnaN, and (iii) its errors are proofread by the 3′ > 5′ exonuclease activity of PolC in a stable template-DnaE–PolC complex. Collectively our data show that protein–protein interactions within the replisome modulate the activity and fidelity of DnaE, and confirm the prominent role of DnaE during *B. subtilis* replication.

## 1. Introduction

A large multi-protein molecular machine, known as the replisome, accurately copies parental genomes during semi-conservative DNA replication. Bacterial replisomes are assembled at a specific chromosomal site known as the chromosomal origin, *oriC*. The strictly conserved initiation protein, DnaA, aided by other species-specific replication initiation proteins, locally melts the parental DNA duplex at *oriC* and mediates anti-parallel loading of two homohexameric ring helicases, one on each of the exposed template strands [1,2]. The remaining replisomal components are then assembled, establishing two fully fledged fork complexes that move away from *oriC* in opposite directions, replicating both the leading and lagging strands. Because of the strict 5′ > 3′ polarity of DNA polymerization and the anti-parallel parental DNA strands, the nascent leading strand is synthesized continuously while the nascent lagging strand is synthesized discontinuously, with DnaG primase repeatedly priming synthesis of Okazaki fragments that are subsequently processed and joined together [3–5]. Duplication of the chromosome is completed when the replication fork complexes meet in the terminus area.

It is now becoming increasingly clear that replisomes diverged to some extent in bacterial taxa, generating replication machineries with different compositions, architectures and *modus operandi* [6,7]. For instance, systematic analyses of sequenced bacterial genomes showed that bacteria often encode more than one DNA polymerase of the C family, which is subdivided into four basic groups: DnaE1, DnaE2, DnaE3 and PolC [8,9]. Polymerases of the DnaE1, DnaE3 and PolC groups are essential for chromosomal and plasmid theta replication [3,10–13], while enzymes of the DnaE2 group are associated with non-essential translesion synthesis in DNA damage tolerance and induced mutagenesis [14,15]. The replicative polymerase of *Escherichia coli*, called the α subunit, belongs to the DnaE1 group of essential replicative polymerases. It assembles with a 3′ > 5′ exonuclease (termed *ɛ* or DnaQ) and the *θ* subunit into a heterotrimer to form the core of the DNA polymerase III holoenzyme endowed with polymerase and 3′ > 5′ proofreading activities [16]. Two or three copies of this complex interact with two to three copies of the DnaX subunit of the clamp loader within the replisome to synthesize simultaneously the leading and lagging strands [17–20].

DnaE3 polymerases are found alongside PolC polymerases in the Gram-positive low G + C content firmicutes [8,9]. *In vitro* studies in model firmicutes (*Bacillus subtilis*, *Streptococcus pyogenes* and *Staphylococcus aureus*) showed that PolC exhibits all the expected features of replicative polymerases; it interacts with DnaX and DnaN (the polymerase clamp), with two PolC molecules interacting with the DnaX complex, has high processivity in complex with DnaN, polymerises DNA in the presence of the DnaX complex and DnaN at a rate similar to *in vivo* replication forks, and is highly accurate because of an internal domain endowed with a 3′ > 5′ exonuclease proofreading activity [21–28]. PolC was also responsible for the bulk of leading and lagging strand synthesis in a fully reconstituted double-stranded, rolling-circle replication assay involving 13 purified proteins [29]. Several studies support the notion that PolC is a major replicative polymerase *in vivo*: (i) cells resistant to 6-(*p*-hydroxyphenylhydrazino)-pyrimidines, a series of related compounds that inhibit PolC activity by competing with the entry of dGTP or dATP in the catalytic site, encode a drug-resistant PolC protein [26,30,31]; (ii) some thermosensitive PolC mutants cause fast arrest of DNA synthesis at restrictive temperature [22]; (iii) mutations that alter either the polymerase or the 3′ > 5′ proofreading catalytic sites of PolC cause a mutator phenotype [21,32]; and (iv) PolC co-localizes dynamically with replisomal proteins at the site of DNA synthesis near mid-cell [10,33,34].

Although PolC fulfils all the requirements for a replicative polymerase, it cannot extend from the 3′-OH ends of RNA primers [29], instead this function is carried out by DnaE [29,35]. The DnaE network of protein interactions with replisomal proteins (illustrated in electronic supplementary material, figure S1) is fully consistent with this role: interactions of DnaE with DnaN, DnaG (the primase), DnaC (the helicase) and HolA (a subunit of the clamp loader termed δ) may couple primer synthesis to DnaN and DnaE loading during lagging strand synthesis [36–42]. Moreover, *in vivo* studies showed that DnaE is mainly involved in lagging strand synthesis and co-localizes with the replication machinery [10]. However, the amount of DNA replicated by DnaE *in vivo* is uncertain: in the fully reconstituted rolling-circle replication assay, DnaE briefly extends RNA primers before handing off DNA synthesis to PolC [29], while in simplified primer extension assays DnaE can synthesize DNA fragments several kilobases long and its processivity and velocity are stimulated by DnaN (≥7.2 kb) and the single-strand DNA binding protein SSB (from 60 up to 240 nt s^−1^), respectively [23,29,35].

DNA polymerases replicate genomes with high accuracy. Replication fidelity is the collective result of three sequential events: (i) selection of the correct nucleotide in the catalytic site of the polymerase and inability for the enzyme to extend mispaired 3′-OH ends; (ii) removal of any misinserted nucleotides at the 3′-OH end of growing chains by a 3′ > 5′ exonuclease proofreading activity; (iii) post-replicative correction of polymerase errors by the mismatch repair system [43]. *Bacillus subtilis* encodes a mismatch repair system [44] and the main PolC polymerase has an internal domain endowed with a 3′ > 5′ proofreading exonuclease activity that corrects its errors [23,27,28,32,35]. By comparison, DnaE has no intrinsic 3′ > 5′ proofreading exonuclease activity [23,27,28,32,35] and *Streptococcus pyogenes* studies showed that, unlike *E. coli* DnaE and DnaQ, the DnaE_Sp_ and DnaQ_Sp_ homologues do not interact with each other to improve the fidelity of DnaE_Sp_ [45]. Moreover, the catalytic site of DnaE from *B. subtilis* and *S. pyogenes* is promiscuous, allowing the enzyme to incorporate and extend mispaired nucleotides at a very high frequency in damaged and native templates [35,39,45]. These properties are incompatible with DnaE playing a substantial role in chromosome replication in *B. subtilis*, as this would result in mutagenesis compromising the fidelity of the genome. However, the error-prone polymerase activity of DnaE is restricted when in complex with other replisomal proteins such as the helicase DnaC and the primase DnaG [39], and DnaE overproduction does not increase the spontaneous mutagenesis rate, as readily observed for error-prone Y-type polymerases [35,46,47]. Hence, it appears that DnaE errors are swiftly prevented and/or corrected during DNA replication *in vivo*.

Here, we further analysed the DnaE polymerase activity and fidelity. Our data collectively indicate that the DnaE polymerase activity is substantially stimulated by other replisomal proteins and becomes sufficiently powerful to synthesize substantial amounts of DNA *in vitro* and *in vivo*, at least in some genetic contexts. We also show that the error-prone activity of DnaE is strongly inhibited when bound to DnaN and that its misincorporations are proofread during DNA replication by the 3′ > 5′ exonuclease activity of PolC *in trans* and further corrected post-replication by the mismatch repair system. Hence DnaE within the replication fork appears to exhibit all the requirements of processivity, power and fidelity for a replicative polymerase.

## 2. Material and methods

### 2.1. Bacterial strains and plasmids

*Bacillus subtilis* strains and plasmids are listed in electronic supplementary material, table S1 along with strategies used for their construction (see below for more details). The *E. coli* strain used for plasmid constructions was DH5α (supE44 supF58 hsdS3 (rB− mB−) dapD8 lacY1 glnV44 Δ(gal-uvrB)47 tyrT58 gyrA29 tonA53 Δ(thyA57)). Cells were grown in LB or in minimal medium [(14 g l^−1^ K_2_HPO_4_, 6 g l^−1^ KH_2_PO_4_, 2 g l^−1^ (NH_4_)_2_SO_4_, 1 g l^−1^ sodium citrate, 0.011 g l^−1^ ferric citrate, 0.004 g l^−1^ FeCl_3_, 2 mM MgSO_4_, 0.2 mM CaCl_2_, 10 µM MnCl_2_, 1 µM FeSO_4_)] supplemented with 0.2% w/v glucose and 0.2% w/v casein acid hydrolysate. The antibiotics used were: ampicillin (50–100 µg ml^−1^); erythromycin (0.6 µg ml^−1^); phleomycin (2 µg ml^−1^); kanamycin (5 µg ml^−1^); chloramphenicol (5 µg ml^−1^); spectinomycin (60 µg ml^−1^); rifampicin (10 µg ml^−1^); HB-EMAU (5 µg ml^−1^).

The pDR111-dnaE plasmid, which contains the *dnaE* gene fused to the IPTG-dependent *Phyper-spank* promoter flanked by the front and back regions of *amyE*, was constructed as follows. First, a PCR product was synthesized from the genomic DNA of the 168 strain. It contained the *dnaE* open reading frame, its ribosome binding site and a *NheI* restriction site at both ends. Upon *NheI* restriction, the fragment was inserted at the *NheI* site of pDR111. Derivatives of pDR111-dnaE containing a mutated form of *dnaE* (D1–3) were constructed as follows: PCR fragments starting from an extremity of the *dnaE* gene and ending at the mutation site were synthesized, fused by combinatory PCR, restricted with *NheI* (this site flanked the *dnaE* gene contained in the PCR fragment) and cloned at the *Nhe*I site of pDR111. This yielded plasmids pDR111-dnaED1, -dnaED2 and -dnaED3. The constructions were validated by DNA sequencing.

To construct strains encoding the WT or mutated (D > A) forms of *dnaE* from the *Phyper-spank* promoter at the chromosomal *amyE* locus, competent cells of the EDJ48 strain were transformed with the DNA of pDR111-type plasmids. Spectinomycin-resistant transformants were selected at 30°C and cells harbouring the transcription fusion as a result of double cross-over between the front and back regions of *amyE* were selected. This yielded strains DGRM821, 824, 825 and 827. To generate strains DGRM836 and 837, the *dnaE*(Ts) mutation of EDJ48 was replaced by a WT or M7 sequence, respectively, by transforming EDJ48 competent cells with PCR products containing *dnaE* or *dnaEM7*. Transformants were selected at 47°C and verified by DNA sequencing. The strain DGRM799 containing the *spc* marker downstream of *polC* was constructed by transforming *B. subtilis* 168 cells with a PCR product as follows. First, the marker resistance gene and two approximately 1.5 kb long DNA fragments containing the 3′ end of *polC* and downstream sequences were amplified using partially complementary oligonucleotides and appropriate templates. Second, a combinatory PCR allowed the production of a fragment containing the resistance gene flanked by the chromosomal segments. Third, the combined fragment was used to transform competent cells and transformants were selected on LB plates supplemented with spectinomycin. A similar strategy was used to construct strains deleted for *dinG*, *kapD*, *yprB*, *ppsA* and *yhaM* (DGRM803, DGRM804, DGRM806, DGRM808 and DGRM810), with the resistance marker replacing the deleted gene. The structure of the strains was confirmed by DNA sequencing. Other strains were constructed by transforming competent cells with genomic or plasmid DNA and appropriate selection on LB plates (see electronic supplementary material, table S1 for details).

### 2.2. Molecular biology

Molecular biology experiments and *E. coli* and *B. subtili*s transformations were carried out using standard procedures. PCR amplifications for strain characterization and DNA cloning were carried out as recommended by suppliers (TaKaRa Ex TaqTM from Takara Shuzo Co., Shiga, Japan and Vent polymerase from New England BioLabs, Hitchin, Hertfordshire, UK, respectively). Oligonucleotides were purchased from Sigma-Aldrich (Evry, Fr) or MWG-Biotech (Ebersberg, Germany) (the sequences are available upon request). DNA sequencing was performed on PCR products by the GATC commercial company or after Exonuclease I, shrimp Alkaline Phosphatase (Amersham France, Les Ulis), terminator sequencing kit treatment (Applied Biosystems PRISM BigDye) on a PerkinElmer 9600 thermal cycler, and were then analysed on an Applied Biosystems 3700 DNA analyser. Genomic DNA was prepared from cells pelleted by centrifugation and resuspended in 0.7 ml of lysis buffer (Tris–HCl pH 8 50 mM, EDTA pH 8 10 mM, NaCl 150 mM and lysozyme 5 mg ml^−1^). After 10 min of incubation at 37°C, cells were treated with sarcosyl buffer (1.2% w/v) for 20 min at 65°C. Peptides and cell fragments were removed by two successive phenol/chloroform treatments. The DNA was then recovered by ethanol precipitation and resuspended in water. Nucleotide concentration was determined with a NanoDrop 2000 Spectrophotometer (ThermoFisher Scientific, Illkrich, France).

### 2.3. Analysis of spontaneous mutagenesis

Strains of interest were streaked on LB and isolated colonies were then grown in the same media and, at saturation, appropriate dilutions were plated on solid LB containing or not 10 µg ml^−1^ rifampicin to count Rif^R^ and viable cells, respectively. To measure the Trp^+^ reversion frequency, the saturating cultures were washed twice in minimal medium supplemented with glucose 2 g l^−1^ before plating on minimal medium containing 15 g l^−1^ agar, 2 g l^−1^ casein hydrolysate (acid hydrolysed) and 2 g l^−1^ glucose to select for Trp^+^ revertants.

### 2.4. Quantitative PCR

Non-replicating cells (stage II sporlets) were prepared as described [48] and genomic DNA was extracted from non-replicating and replicating cells (i.e. cells growing exponentially at OD_600 nm_ = 0.2), as indicated above. To measure the *ori/ter* ratio, primers mapping in the *oriC* region (in *dnaA*: LJ114: TCCGAGATAATAAAGCCGTCGA; LJ115: CTGGGTTTGTTCTTTCCCCG) and terC (in yoxD: BD280: TTTCAGTTGTGCCACCATGT; BD281: ATTTGCCGTTCTCGGGTTA) regions were used at 200 nM in 12 µl of the 1× Absolute Bleu SYBR Green ROX Mix (Thermo Scientific, Surrey, UK) or the SYBR Premix Ex TaqTM Tli RNaseH Plus (Takara, Shiga, Japan) containing approximately 10 000 to 100 copies of the chromosome (obtained by successive half dilutions). Replicating and non-replicating DNAs were analysed simultaneously in duplicates. The ΔCq were determined automatically from the cycle threshold. Primer efficiency ranged from 97% to 103% and was controlled during every qPCR analysis. The *ori/ter* ratio of replicating DNAs was normalized using the corresponding *ori/ter* ratio of non-replicating sporlets. The qPCR analyses were carried out on a Mastercycler ep realplex (Eppendorf, Le Pecq, France).

### 2.5. Cloning, expression, purification and quantification of WT and mutated DNA polymerases

Expression and purification of *B. subtilis* SSB, DnaN (β clamp), DnaX (τ subunit), YqeN (δ) and HolB (δ)′ were carried out as described elsewhere [49–51].

The *B. subtilis dnaE* gene was cloned, using Ligation Independent [52], into the p2CT plasmid (AmpR; a kind gift from James Berger) to construct the p2CT-NT-HT-DnaE expression vector coding for DnaE protein with an N-terminal hexahistidine-tag maltose-binding-tag removable with TEV protease cleavage. The DnaE_pol-_ (D382A/D384A) mutation was introduced using this vector to construct the p2CT-NT-HT-DnaE_pol-_ expression vector. The mutation was inserted using the Q5 Site-Directed Mutagenesis kit (New England Biolabs) with the mutagenic primers 5′-GCTATTGACTTTCCCGATACTAGAAGGGATG-3′, 5′-ATAGCCGGCATGCTGACGCGTT-3′. All DnaE proteins were expressed and purified as follows. The appropriate expression vector was transformed into BL21 (DE3) Rosetta *E. coli.* The cells were cultured in 2xYT media with 50 µg ml^−1^ carbenicillin and 1% w/v glucose at 37°C in a shaking incubator (180 rpm) until mid-log phase (OD595 = 0.8) followed by incubation at 4°C for 10 min, prior to induction of protein expression by the addition of 1 mM IPTG (isopropyl-thiogalacto-pyranoside). Protein expression was allowed to proceed for 3 h at 25°C. The cell pellet was harvested by centrifugation at 3000*g* for 15 min at 4°C and resuspended in 30 ml sonication buffer (20 mM HEPES-NaOH pH 8.0, 0.5 M NaCl, 20 mM imidazole, 10% (v/v) glycerol, 1 mM PMSF, protease inhibitor cocktail VII (Fischer)). Cells were disrupted by sonication and the soluble lysate supernatant was collected by centrifugation at 30 000*g*, 30 min at 4°C, filtered through 0.22 µm filters and loaded onto a HisTrap HP column (GE Healthcare) equilibrated in 20 mM HEPES-NaOH pH 8.0, 0.5 M NaCl, 20 mM imidazole and 10% (v/v) glycerol. His-tagged DnaE was eluted with a 15-column volume gradient of 0–0.5 M imidazole in the same buffer. Protein containing fractions were pooled, TEV protease was added at 1 : 15 (protein : TEV) mass ratio followed by dialysis against 20 mM HEPES-NaOH pH 8.0, 2 M NaCl, 0.5 M urea and 10% (v/v) glycerol at 4°C. The dialysed protein sample was then filtered (0.22 µm filters) and loaded onto a HisTrap HP column equilibrated in the same buffer, and the flow through was collected. Solid ammonium sulfate was added at 4°C to final concentration of 55% (w/v). Precipitated protein was collected by centrifugation at 4000*g*, 15 min, 4°C, suspended in 20 mM HEPES-NaOH pH 8.0, 50 mM NaCl, 1 mM DTT, 10% (v/v) glycerol and loaded onto a HiTrap Q HP column (GE Healthcare) equilibrated in the same buffer, keeping the conductivity below 4 mS. The protein was eluted with a 10 column volume of 0–1 M NaCl gradient in 20 mM HEPES-NaOH pH 8.0, dialysed overnight against 20 mM HEPES-NaOH pH 7.5, 250 mM NaCl, 1 mM DTT, 10% (v/v) glycerol, aliquoted, frozen in liquid nitrogen and stored at −80°C. Purity (less than 98%) was assessed by SDS-PAGE and quantification was carried out spectrophotometrically using the extinction coefficient 87 030 M^−1^ cm^−1^ for DnaE.

The *B. subtilis polC* gene was cloned, using Ligation Independent Cloning [52], into the p2BT plasmid (AmpR; a kind gift from James Berger) to construct the p2BT-NT-HT-PolC expression vector coding for a PolC protein with an N-terminal His-tag removable with TEV protease cleavage. The PolC_pol-_ (D966A/D968A) and PolC_exo-_ (D425A/E427A) mutations were introduced using this vector to construct the p2BT-NT-HT-PolC_pol-_ and p2BT-NT-HT-PolC_exo-_, respectively. The mutations were inserted using the Q5 Site-Directed Mutagenesis kit (New England Biolabs) with the mutagenic primers 5′-CGCTTTGAACTTCTCAGGGGAATATC-3′, 5′-ATAGCAGGTACTTTGTCCCCTTTAAATC-3′ for PolC_pol-_ (D966A/D968A) and 5′-TGCGACGACAGGATTGTCTGCTG-3′, 5′-ACAGCAAAAACAACATATGTTTCTTCTTCG- 3′ for PolC_exo-_ (D425A/E427A). All PolC proteins were expressed and purified as follows. The appropriate expression vector was transformed into BLR(DE3) *E. coli*. Culturing, expression and cell harvesting were carried out as described for DnaE (see above). The harvested cell pellet was resuspended in 30 ml sonication buffer (20 mM HEPES-NaOH pH 7.5, 0.5 M NaCl, 20 mM imidazole, 10% (v/v) glycerol, 1 mM PMSF, protease inhibitor cocktail VII (Fisher)). The cells were disrupted by sonication, the soluble lysate was collected by centrifugation, as described for DnaE, filtered through 0.22 µM filters and loaded onto a HisTrap HP column (GE Healthcare) equilibrated in 20 mM HEPES-NaOH pH 7.5, 0.5 M NaCl, 20 mM imidazole and 10% (v/v) glycerol. His-tagged PolC proteins were eluted with a 15-column volume gradient of 0–0.5 M imidazole in the same buffer. Protein containing fractions were pooled, TEV protease was added at 1 : 20 (protein : TEV) mass ratio followed by dialysis against 20 mM HEPES-NaOH pH 7.5, 50 mM NaCl, 10% (v/v) glycerol at 4°C . The dialysed protein sample was then loaded onto a HisTrap HP column equilibrated in 20 mM HEPES-NaOH pH 7.5, 50 mM NaCl, 10% (v/v) glycerol and the flow through was collected and loaded onto a HiTrap Q HP column (GE Healthcare) equilibrated in 20 mM HEPEs-NaOH pH 7.5, 50 mM NaCl, 1 mM DTT, 10% (v/v) glycerol. PolC proteins were eluted with a 15 column volume gradient 50–1000 mM NaCl in the same buffer. Fractions containing PolC protein were pooled and loaded onto a HiLoad 26/60 Superdex-200 column equilibrated in 20 mM HEPES-NaOH pH 7.5, 200 mM NaCl, 1 mM DTT, 10% (v/v) glycerol. PolC containing fractions were pooled, aliquoted, frozen in liquid nitrogen and stored at −80°C. Purity (less than 98%) was assessed by SDS-PAGE and quantification was carried out spectrophotometrically using the extinction coefficient 130 835 M^−1^ cm^−1^ for PolC. All the purified proteins (PolC, PolC_pol-_, PolC_exo-_, DnaE, DnaN, DnaX, HolB, YqeN and SSB) are shown in electronic supplementary material, figure S2.

### 2.6. Primer extension assays with short oligonucleotide template

DnaE activity was investigated with primer extension assays using a short (15mer) radiolabelled synthetic oligonucleotide, either RNA (5′-AAGGGGGUGUGUGUG-3′) or DNA (5′-AAGGGGGTGTGTGTG-3′), annealed onto a longer (110mer) template (5′-CACACACACACACACACACACACACACACACACACA CACACACACACACA**CACACACACCCCCTTT**AAAAAAAAAAAAAAAAGCCAAAAGCAGTGCCAAGCTTGCATGCC-3′) to produce a substrate with a 45 nt 3′-overhang and a 50 nt 5′-overhang. The bold underlined sequence indicates the annealed double-stranded region. In primer extension reactions, the polymerases extended the short oligonucleotide from its 3′-OH end, copying the 5′-overhang, to produce a final product 65 nt long. Assays were carried out in 20 mM HEPES pH 7.5, 50 mM NaCl, 10 mM MgCl_2_, 1 mM DTT and 200 µM dNTPs. The reaction mixture containing the buffer, dNTPs and the polymerase (different concentrations of DnaE as indicated) was pre-incubated at 37°C for 5 min before the addition of radiolabelled substrate (0.66 nM) to initiate the reaction. The reaction proceeded for 15 min, as indicated, at 37°C and terminated by the addition of 1/5th 5× Urea-Stop buffer (5 mM Tris–HCl pH 7.5, 20 mM EDTA, 7.5 M urea). The mixture was then heated to 95°C for 2 min and analysed by electrophoresis through 15% (v/v) polyacrylamide–urea sequencing gels. Gels were dried under vacuum and imaged using a phosphorimager and associated software (Bio-Rad). Data were analysed and plotted using GraphPad Prism 6.

The activities of PolC, PolC_pol-_ and PolC_exo-_ were investigated as above with minor modifications: the titration assays were carried out for 15 min at 37°C (the different protein concentrations were as indicated) while the time point assays were carried out with 80 nM PolC.

### 2.7. Electrophoretic mobility shift assays (EMSA)

Polymerase binding to a 30mer DNA oligonucleotide template (5′-ACACACACACACACACACACACACCCCCTT-3′) primed by a shorter radiolabelled 15mer DNA (5′-AAGGGGGTGTGTGTG-3′) or RNA (5′- AAGGGGGUGUGUGUG-3′) primer or paired with a 15mer DNA primer with a A:G mismatch at the 3′-OH end (5′-AAGGGGGTGTGTGTA-3′) was investigated with EMSA. Binding reactions with the fully paired primers were carried out in a total volume of 20 µl in 20 mM HEPES pH 7.5, 50 mM NaCl, 10 mM MgCl_2_ and 1 mM DTT. Reaction mixtures containing the buffer and the protein at different concentrations, as indicated, were pre-incubated at 37°C for 5 min before the addition of the appropriate radiolabelled DNA substrate (0.66 nM) and further incubation at 37°C for 5 min. The effect of increasing concentrations of PolC or PolC_exo-_ (25, 50, 100, 250, 500 and 1000 nM) on DnaE complexes with the 3′-mismatch substrate was investigated in a total volume of 15 µl in 20 mM HEPES pH 7.5, 50 mM NaCl, 10 mM MgCl_2_ and 1 mM DTT. Reaction mixtures containing the buffer, the DNA substrate (0.66 nM) and DnaE (1 µM) were pre-incubated at 37°C for 5 min before the addition of increasing concentrations of PolC or PolC_exo-_ and further incubation at 37°C for 5 min. After the addition of gel loading dye (1/5th of 5× loading dye; 50% (v/v) glycerol, 0.15% (w/v) bromophenol blue, 0.25% (w/v) xylene cyanol) all samples were analysed by PAGE through 5% (v/v) native polyacrylamide gels. Gels were dried under vacuum and imaged using a molecular imager and associated software (Bio-Rad).

### 2.8. Primer extension assays with long ssM13mp18 template

Primer extension assays with ssM13mp18 (7249 nt) (Affymetrix) template were carried out by annealing a single radiolabelled RNA (5′-CAGUGCCAAGCUU GCAUGCC-3′) or DNA (5′-CAGTGCCAAGCTTGCATGCC-3′) primer and monitoring polymerase-mediated extension (nascent DNA synthesis) at 37°C in the absence or presence of SSB and with or without DnaN (β) plus DnaX (τ), HolB (δ′) and YqeN (δ) by alkaline agarose electrophoresis, as indicated. Assays were carried out in a total volume of 105 µl and 15 µl samples were taken at time intervals, inactivated by the addition of 50 mM EDTA and 1/6th alkaline agarose loading dye (300 mM NaOH, 6 mM EDTA, 18% (v/v) glycerol, 0.15% (w/v) bromophenol blue, 0.25% (w/v) xylene cyanol), heated to 95°C for 2 min and then analysed by alkaline agarose electrophoresis, as indicated. Alkaline agarose gels were dried overnight and imaged using a phosphorimager and associated software (Bio-Rad). Typically, the reaction buffer contained 20 mM HEPES pH 7.5, 50 mM NaCl, 10 mM MgCl_2_, 1 mM DTT, 500 µM dNTPs, 2.5 mM ATP and 2 nM radioactively labelled primed ssM13mp18 template. In all reactions with SSB-coated templates the reaction buffer was pre-incubated with SSB (1 µM), DnaN (80 nM), DnaX (120 nM), HolB (40 nM) and YqeN (40 nM) at 37°C for 5 min before the addition of the polymerase DnaE (80 nM) to initiate the reactions. Quantification of percentage nascent DNA synthesized was carried out using a molecular imager and associated software (Bio-Rad) and bar graphs were prepared using GraphPad Prism 4.

### 2.9. Exonuclease assays

PolC exonuclease assays were carried out in a total volume of 50 µl using a DNA substrate (2 nM) comprising a radioactively labelled 20mer synthetic DNA oligomer (5′-CAGTGCCAAGCTTGCATGCC-3′) annealed onto ssM13mp18 and pre-incubated with 80 nM DnaE for 5 min at 37°C in 20 mM HEPES pH 7.5, 50 mM NaCl, 10 mM MgCl_2_, 1 mM DTT before adding the PolC or PolC_exo-_ (80 nM) and incubating at 37°C for a further 15 min. The reaction was terminated with boiling at 95°C for 5 min before resolving the reaction products through a 15% urea/acrylamide sequencing gel.

### 2.10. Error-prone DnaE polymerase assays

The error-prone DnaE polymerase activity was assayed as described for the primer extension assays with ssM13mp18 template, omitting dGTP from the reaction mixture to force DnaE errors at cytosine positions along the template. The concentrations of the proteins were 360 nM DnaE, 360 nM PolC, 180 nM YqeN, 180 nM HolB, 540 nM DnaX and 360 nM DnaN.

## 3. Results

### 3.1. DnaE is required for both replication initiation and elongation

**Figure 1.**
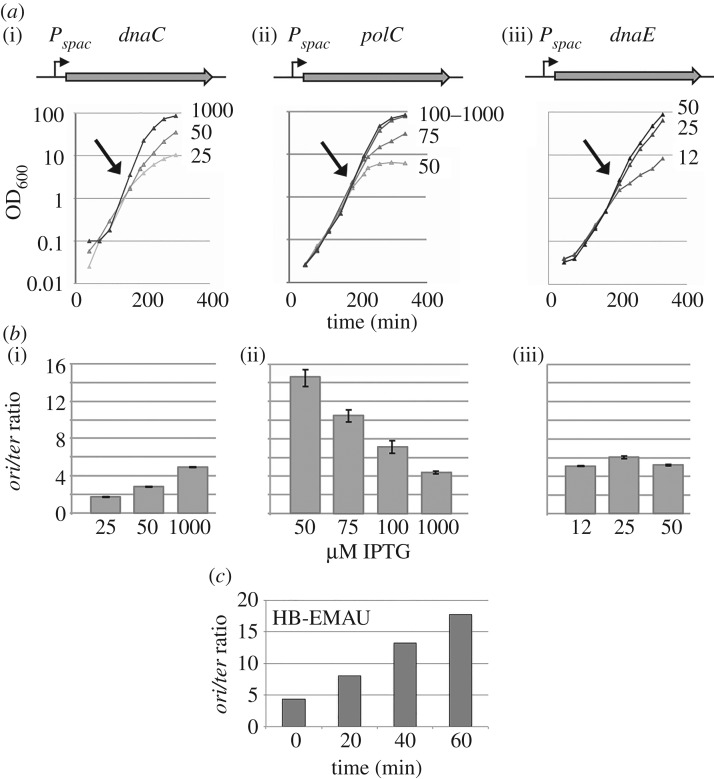
DnaE is required for both initiation and elongation of replication. Strains HVS597, HVS609p and HVS614p encode *dnaC*, *polC* or *dnaE* from the *Pspac* IPTG-inducible promoter. Strains were grown overnight at moderate inducer concentration and then were diluted and maintained in exponential phase (by serial dilutions) in the presence of the indicated IPTG concentrations (μM). Cell growth was monitored spectrophotometrically (*a*) and *the ori/ter* ratio was measured by qPCR (*b*). The ratio was determined just before the growth rate decrease/arrest (arrows in (*a*)). At these time points, cells suffer from a dramatic replication defect for about 2–3 generations. Bars represent mean values with standard errors of at least three independent cultures. (*c*) Exponentially growing wild-type cells (168) were treated with a lethal concentration of a HB-EMAU (10 µM). The *ori/ter* ratio was determined by qPCR at different time points upon HB-EMAU treatment. A representative experiment is shown.

Cells growing fast undergo overlapping replication cycles that result in a higher concentration of origin versus terminus DNA sequences. It is well documented that this *ori/ter* ratio varies in replication mutants; for instance, it increases in elongation mutants and decreases in cells defective in replication initiation. To gain insights into the biological function of DnaE in DNA replication, we measured the *ori/ter* ratio by qPCR in cells depleted for DnaE, PolC or DnaC (the helicase). We used strains encoding the replication genes from the IPTG-inducible promoter *Pspac* (figure 1*a*) and grew the cells in LB in the presence of various concentrations of inducer. Although depletion of DnaE, PolC and DnaC caused similar growth inhibition (figure 1*a*), the *ori/ter* ratio was affected in different ways (figure 1*b*). In DnaC-depleted cells, the ratio decreased from 4.9 to 1.7 (figure 1*b*(i)). This decrease is consistent with DnaC being an early initiation enzyme operating in a highly processive manner during elongation. At low DnaC concentrations the potential for initiation decreases, reducing fork formation and DNA synthesis at the origin, while on-going replication forks continue to progress efficiently all along the genome to ultimately duplicate the terminus region. These combined responses result in an overall decrease of the *ori/ter* ratio.

By comparison, PolC depletion caused a marked increase in the *ori/ter* ratio (from 4.4 to about 15; figure 1*b*(ii)). This increase probably results from two cumulative events: (i) fork speed inhibition (or fork arrest) that delays (or precludes) replication of the terminus region, and hence increases the *ori/ter* ratio (this is consistent with the established role of PolC in DNA elongation); and (ii) over-replication of the *oriC* region in response to a feedback mechanism that may stimulate initiation when elongation is inhibited. Given that the marked increase still occurs in cells lacking Pol I (electronic supplementary material, figure S3, left panel), over-initiation at *oriC* in PolC-depleted cells is probably due to DnaE activity.

In contrast to DnaC and PolC, DnaE depletion had no effect on the *ori/ter* ratio (figure 1*b*(iii)). Given that DnaE depletion inhibits DNA synthesis [10], this result suggests that, in addition to elongation, DnaE is involved in initiation, and acts distributively during elongation. Interestingly, this phenotype occurs in the presence of physiological concentration of PolC. Hence, PolC cannot synthesize DNA at *oriC* under conditions of strong DnaE depletion, or does so for less than 2 kb away from the origin, because the *oriC* primers used in the qPCR assays map 2 kb away from the replication initiation site.

To further investigate the involvement of PolC and DnaE in replication, we next analysed the *ori/ter* ratio in cells treated with lethal concentrations of HB-EMAU (10 µg ml^−1^), a nucleotide analogue that specifically inhibits PolC polymerase activity by trapping the enzyme at elongating 3′-OH ends [26,30] without affecting the DnaE polymerase activity [28,29,53]. We found that HB-EMAU, similar to PolC depletion, caused a marked increase in the *ori/ter* ratio in a Pol I-independent manner (figure 1*c*; electronic supplementary material, figure S3, right panel). This confirms that DnaE is involved in initiation and leads us to hypothesize that over-replication of *oriC* in PolC-compromised cells mainly depends on extension by DnaE of RNA primers synthesized at *oriC* by the primase rather than on illegitimate DnaE extension of 3′-OH ends resulting from stalling of PolC during elongation, as these ends are trapped by HB-EMAU in an inactive complex with PolC.

To provide further evidence for the involvement of DnaE in both initiation and elongation, we analysed replication arrest in nine thermosensitive *dnaE* strains at restrictive temperature (49°C). These strains contain different mutations in the *dnaE* gene that probably arrest replication in different ways [54]. Replication was assessed at various time points upon temperature increase by measuring the *ori/ter* ratio. We identified two groups of thermosensitive mutants (electronic supplementary material, figure S4). In the first group, the *ori/ter* ratio dropped dramatically from 4 to less than 2. These correspond to initiation mutants, as similar phenotypes were observed in the *dnaD23* and *dnaI2* mutants which encode thermosensitive proteins required for helicase loading at *oriC* during initiation. In the second group (including the *dnaE2.10* allele), a less dramatic drop in the *ori/ter* ratio was observed (from 4 to 2.5–3) (electronic supplementary material, figure S4). This moderate decrease indicates that the corresponding mutations affected DNA elongation as observed previously for the *dnaE2.10* thermosensitive mutation [10].

Collectively, these data suggest that DnaE is required for both initiation and elongation of DNA replication, that DnaE is functionally loaded at *oriC* before PolC, and that DnaE-dependent DNA synthesis at *oriC* is mandatory for subsequent PolC-dependent replication.

### 3.2. DnaE is required at the replication fork to synthesize nascent DNA

**Figure 2.**
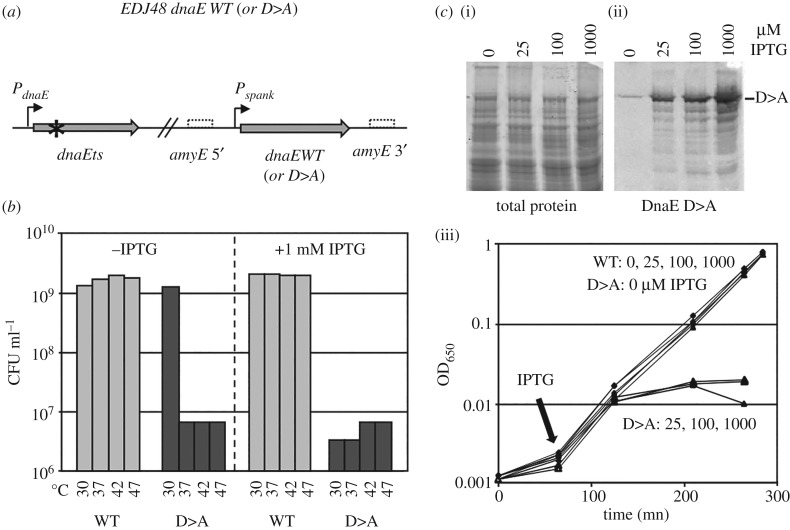
DnaE is required at the replication fork to synthesize nascent DNA. (*a*) Schematic representation of the tested strains. Strains DGRM821, 824–825 and 827 encode the DnaE2.6 thermosensitive form of DnaE from the natural locus and a WT or inactive (D > A) form of DnaE from the IPTG-inducible promoter *Pspank* at the *amyE* locus. Star: Ts mutation; dotted boxes: 5′ and 3′ regions of the inactivated *amyE* locus. (*b*) Complementation assay. Strains were grown in LB at 30°C and plated on a solid broth in the presence and absence of 1 mM IPTG, as indicated. The concentration of cell forming unit (CFU ml^−1^) was determined at various temperatures, as indicated. (*c*) The ectopic copy of *dnaE* was fused to a tag (SPA) at the C-terminus (DGRM830-833) for Western blot detection. Expression of the DnaED > A protein was examined by Western blot, 2 h after IPTG addition (ii), and total proteins were visualized using Sypro red staining (i). (iii) IPTG dependent accumulation DnaED > A and its effect on cell growth at 30°C. Cells were grown exponentially in LB at 30°C. At OD_600 nm_ of approximately 0.002, IPTG (0, 25, 200 and 1,000 µM) was added and cell growth was followed spectrophotometrically. The three DnaED > A mutated strains gave similar results (presented data are with the *dnaED382A* allele).

To determine whether DnaE is required in the replisome for catalytic or structural purposes, the fate of cells encoding DnaE_pol-_ mutant proteins was analysed. The catalytic site of DNA polymerases contains three highly conserved Asp (D) residues and replacement of any one of these residues to Ala (A) abolishes (or strongly reduces) the polymerase activity in *E. coli* DnaE [55,56]. EMBOSS-Water [57] sequence alignment of *E. coli* DnaE with *B. subtilis* DnaE identified the polymerase catalytic site residues in the *B. subtilis* enzyme as D382, D384 and D535 (electronic supplementary material, figure S5). We found that the D > A mutated forms of DnaE at positions 382, 384 or 535 are unable to support cell growth (figure 2). Four strains were constructed (figure 2*a*) and named generically EDJ48 Pspank::dnaE WT or D > A. They encode a wild-type or a D > A variant of DnaE at the *amyE* locus from an IPTG-dependent (*Phyper-spank*) promoter and a thermosensitive (Ts) DnaE protein (DnaE2.6) from its endogenous locus. The Ts protein is functional at 30°C and inactive above 37°C [54]. At restrictive temperatures (37–47°C), the strains encoding both the Ts and native proteins were fully viable, even in the absence of IPTG (figure 2*b*), demonstrating that the heat-inactivated Ts protein does not exert a dominant-negative effect. The viability in the absence of IPTG is likely to be due to the leakiness of the inducible promoter and to the fact that *B. subtilis* survival only needs approximately 30 DnaE molecules per cell (a cell normally contains approx. 350 copies of the enzyme which is likely to carry out both replication and repair functions [35,37,58]). In contrast to the data obtained in the *dnaETs Pspank::dnaEWT* context, none of the isogenic DnaED > A strains were viable at high temperature (the D382A data are presented in figure 2*b*; similar results were obtained with the remaining D > A strains). Moreover, in contrast to native DnaE, a moderate accumulation of DnaED > A in the *dnaETs* context induced filament formation and eventually growth arrest at 30°C (the D382A data are presented in figure 2*c*; similar results were obtained with the remaining D > A strains).

Collectively, these data indicate that the polymerase-deficient D > A forms of DnaE can enter the replisome to compete with the Ts protein for the 3′-OH ends and poison DNA synthesis, a dramatic event that eventually leads to formation of filaments and growth arrest. Support for the fact that the D > A mutants are inactive *in vivo* even at 30°C was further provided using a genetic assay, whereby random gene inactivation was carried out during transformation of competent cells with an integrative plasmid carrying an internal segment of *dnaE*. This assay revealed that plasmid integration occurred with equal efficiency at 30°C in either copy of *dnaE* (i.e. in the Ts or wild-type gene) in strain EDJ48 Pspank::dnaEWT and exclusively in the *dnaED > A* gene in strain EDJ48 Pspank::dnaED > A (data not shown). This latter observation shows that cells encoding the DnaED > A protein and lacking the DnaETs enzyme are not viable. Collectively, our data indicate that the catalytic polymerase activity of DnaE is essential during DNA synthesis. Therefore, the role of DnaE is not limited to allosteric structural effects within the replisome.

### 3.3. Characterization of DnaE with primer extension assays and EMSAs

**Figure 3.**
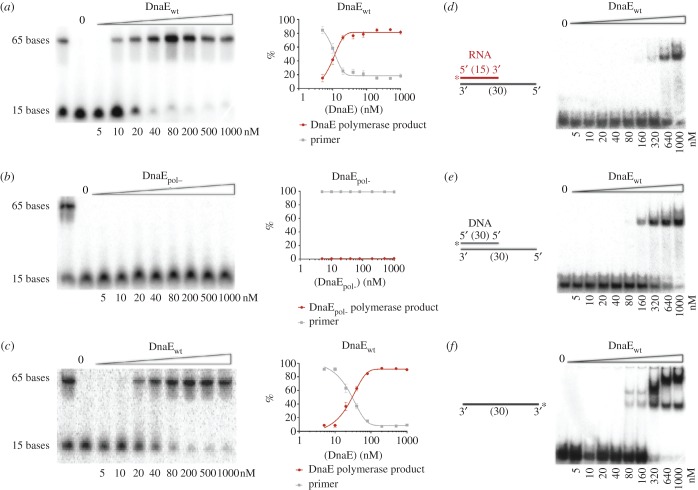
DnaE and DnaE_pol-_ activity assays on short templates. DNA primer (*a*,*b*) or RNA primer (*c*) extension assays using short radiolabelled deoxyribonucleotide or ribonucleotide (15 nt) annealed onto longer oligonucleotide template (110 nt) for the detection of DnaE activity. These were performed using protein concentration titration assays (5, 10, 20, 40, 80, 200, 500 and 1000 nM) with either DnaEWT (*a*,*c*) or DnaE_pol__-_ mutant protein (*b*). The reaction samples were incubated at 37°C for 15 min, resolved by electrophoresis on 15% (v/v) urea gel PAG, the results were analysed using molecular imager and associated software (Bio-Rad) and the percentage of the assay products was plotted using GraphPad Prism 6. Bars represent mean values with standard errors of at least three independent protein samples. (*d*–*f*) EMSAs showing DnaE binding to different radiolabelled nucleotide substrates. The reaction mixture of buffer and substrate (0.66 nM) was pre-incubated at 37°C for 5 min before addition of DnaE at different concentrations, as indicated, and further incubation at 37°C for 5 min. Samples were mixed with loading buffer and analysed through 5% (v/v) native polyacrylamide gels. Lanes labelled 0 show the control radiolabelled substrate on its own. Asterisks indicate the radiolabel position at the 5′-P end.

Biochemical characterization of DnaE was carried out using primer extension assays on a short 110mer DNA template primed by a 15mer ssDNA primer, using wild-type and mutant proteins compromised in polymerase activity. Residues D382 and D384 were mutated to alanines to engineer the DnaE_pol-_ (D382A/D384A) mutant protein. Purified wild-type *B. subtili*s DnaE extends DNA primers (figure 3*a*), whereas DnaE_pol-_ showed no such activity, as expected (figure 3*b*). Neither protein has exonuclease activity (figure 3*a*,*b*), confirming the absence of proofreading activity in DnaE. DnaE was able to extend an RNA primer on a DNA template (figure 3*c*), as expected from previous studies [29,35]. These data demonstrate DnaE activity in the absence of auxiliary proteins in a minimal *in vitro* system with the primed template and the protein. Additionally, we confirm that mutations D382A and D384A abolish polymerase activity in *B. subtilis* DnaE.

EMSAs revealed that DnaE has a slight preference for DNA-primed substrate over RNA-primed template (figure 3*d*,*e*). Multiple shifted bands were observed on a ssDNA 30mer (figure 3*f*), indicating multiple DnaE molecules interacting with the substrate, similarly to all polymerases containing the C-terminal OB fold domain [59,60].

### 3.4. Effect of DnaN and SSB on DnaE activity

**Figure 4.**
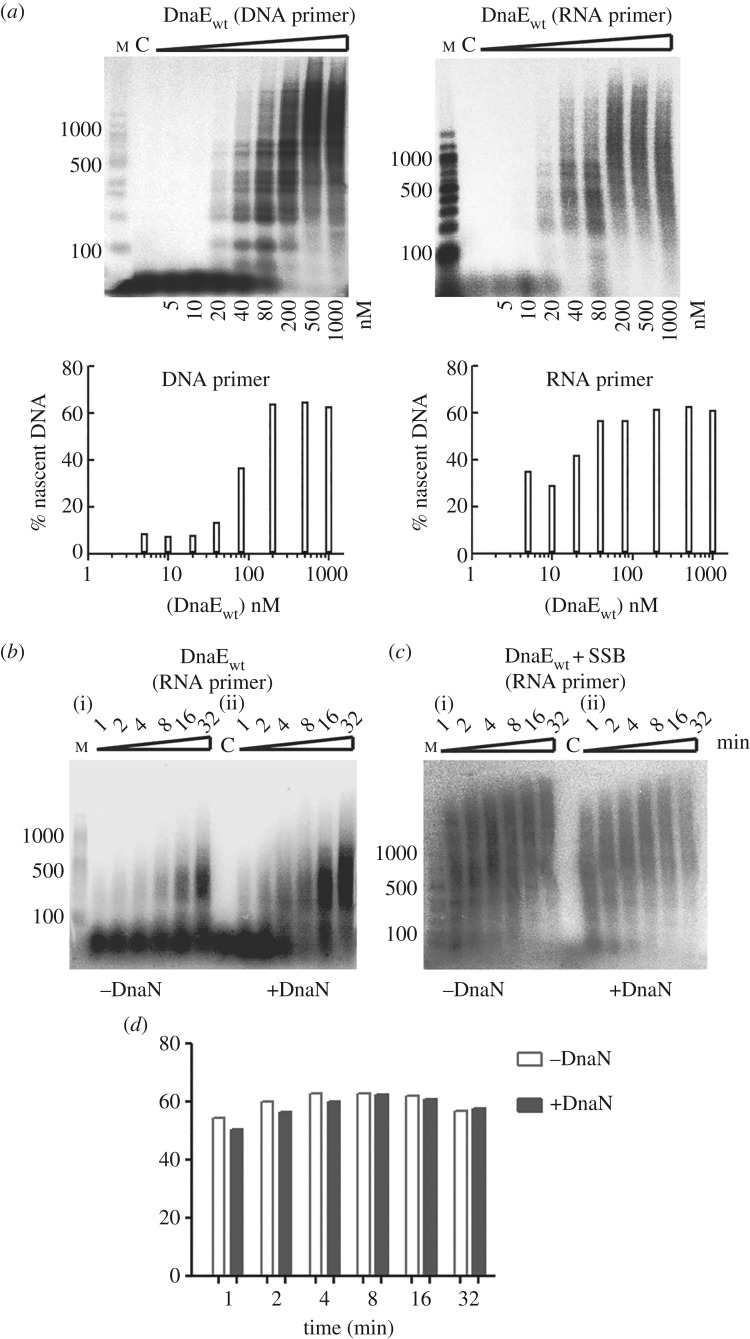
DnaE primer extension assays on a long template and DnaE stimulation by DnaN and SSB. Assays were carried out on M13mp18 template (2 nM) with DnaE in the presence and absence of the clamp DnaN (80 nM) and subunits of the clamp loader: DnaX (120 nM), HolB (40 nM), YqeN (40 nM). (*a*) Primer extension assays at different DnaE concentrations (5, 10, 20, 40, 80, 200, 500 and 1000 nM) primed with DNA and RNA primers, as indicated. Reaction mixtures were incubated at 37°C for 32 min and terminated by the addition of loading dye and incubation at 95°C for 5 min. Quantifications of DnaE-mediated percentage of nascent DNA synthesis is shown underneath each gel. (*b*,*c*) Time course reactions using 80 nM DnaE and an RNA primer in the presence or absence of DnaN and/or SSB (1 µM) as indicated. Reactions were incubated at 37°C for the indicated time and terminated by addition of loading dye and incubation at 95°C for 5 min. Reaction products were analysed through 1.5% (w/v) alkaline agarose gels. Lanes labelled M and C show molecular weight markers and a control reaction without polymerases, respectively. All assays were carried out in triplicates and the gels shown here are representative. (*d*) Quantifications of the primer extension reactions shown in (*c*).

To establish how much nascent DNA can be formed by DnaE and to investigate the effect of the DnaN clamp and SSB on DnaE activity, primer extension assays were carried out using ssM13mp18 DNA (approx. 7.2 kb) template primed either by an RNA or DNA primer. In titration assays, DnaE alone extends both RNA- and DNA-primed templates, with slightly better efficiency in the former case, creating products greater than 1500 bp long (figure 4*a*). In time course reactions with RNA-primed templates, 80 nM of DnaE synthesized nascent DNA fragments up to approximately 300–500 bp (figure 4*b*(i)). This is consistent with previously reported studies on *B. subtilis* and *S. pyogenes* DnaE [23,35,39].

In time course analyses using RNA primed ssM13mp18 DNA, DnaN increased substantially the amount of nascent DNA, while the lengths of the DNA fragments were only marginally increased (figure 4*b*(ii)). Since ssDNA templates *in vivo* are covered by SSB, we carried out similar polymerase assays using ssM13mp18 DNA templates coated with SSB. The template (2 nM) was incubated with SSB (1 µM tetramer; equivalent to 500× molar excess of SSB tetramer over the template, i.e. one SSB tetramer per approx. 14 nt) prior to the addition of polymerases in the presence and absence of DnaN. Under these conditions, there is more than enough SSB to coat the entire ssM13mp18 DNA template [50]. In the presence of SSB, the activity of DnaE was stimulated substantially, producing large amounts of nascent DNA greater than 1500 nt in 1 min (figure 4*c*(i)). Adding DnaN did not change this activity (figure 4*c*—compare (i) and (ii), and the quantification bar graph in figure 4*d*). These data show that DnaE activity is stimulated by DnaN and SSB, consistent with previous observations [23,29,35]. It is shown below that DnaE activity is also stimulated by PolC.

### 3.5. DnaE and PolC misincorporations are cumulative and corrected by MutSL

The *in vitro* results presented above indicate that *in vivo*, DnaE can replicate more than a few nucleotides downstream of an RNA primer. If so, its misincorporations during DNA elongation may be corrected by the mismatch repair system [61], and cells encoding mutator forms of both DnaE and PolC may express a mutagenesis rate higher than single PolC mutants. To test this hypothesis, we compared the rates of spontaneous mutagenesis in cells proficient or deficient in the mismatch repair system and encoding a WT or a mutator form of DnaE, PolC or DnaE and PolC. The *polC25* (L1177 W) and *polC27* (F1264S) mutations were previously shown to confer a mutator phenotype [21,32]. We introduced the E588 K mutation in DnaE to yield the DnaEM7 strain. The corresponding mutation E612 K in *E. coli* DnaE strongly stimulates the rate of spontaneous mutagenesis [62,63]. Mutagenesis was assessed using the rifampin resistance (Rif^R^) and the Trp^+^ reversion assays. The former assay allows the detection of base substitutions in a few amino acids of the β subunit of the RNA polymerase [64], whereas the latter detects +1 frameshifts in a tract of five adenines in *trpC* (P. Noirot 2005, personal communication).

**Figure 5.**
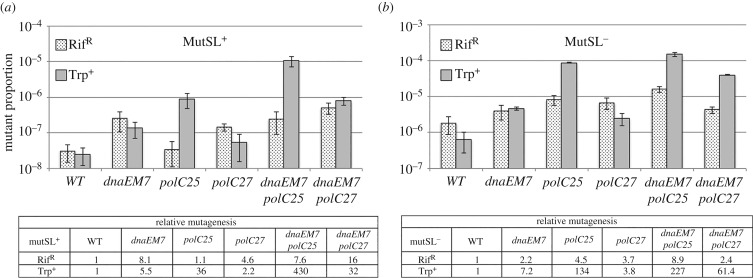
DnaE and PolC misincorporations are cumulative and corrected by MutSL. Six isolated colonies of isogenic TrpC^−^ strains encoding a WT or a mutated form of DnaE, PolC or DnaE and PolC, and proficient or deficient for the mismatch repair system, were cultivated in LB and plated to measure the proportion of Rif^R^ and Trp^+^ cells. The relative proportions of mutants are given in the tables. (*a*) MutSL^+^ context: WT (JJS9); *dnaEM7* (DGRM850), *polC25* (DGRM855), *polC27* (DGRM857), *dnaEM7 polC25* (DGRM860) and *dnaEM7 polC27* (DGRM861). (*b*) MutSL^−^ context: WT (DGRM812); *dnaEM7* (DGRM871), *polC25* (DGRM873), *polC27* (DGRM874), *dnaEM7 polC25* (DGRM875) and *dnaEM7 polC27* (DGRM876). The proportion of spontaneous mutants in the reference strains are: MutSL^+^ context: Rif^R^: 3.1 × 10^−8^ ± 1.6 × 10^−8^; Trp^+^: 2.5 × 10^−8^ ± 1.3 × 10^−8^: MutSL^−^ context: Rif^R^: 1.8 × 10^−6^ ± 9.3 × 10^−7^; Trp^+^: 6.5 × 10^−7^ +/- 3.9 × 10^−7^. Bars represent mean values and standard errors.

Our results in the MutSL^+^ background showed a slight (1–8 times) increase in mutagenesis in PolC- and DnaE-mutated cells over the wild-type background (figure 5*a*), with the exception of *polC25* that exhibited a stronger (36-fold) mutation rate in the Trp^+^ assay. Importantly, the mutagenesis rate in the *polC25 dnaEM7* and *polC27 dnaEM7* strains were 3–15 times higher compared with parental PolC mutants. In the absence of the mismatch repair system, an approximately 50-fold increase in mutagenesis was observed in all the tested strains and the cumulative effect in *polC dnaE* double mutants was still present although to a lesser extent (figure 5*b*). Note that the mutagenesis rates in replication mutants compared with that of wild-type strains (the relative mutagenesis rate; see table in figure 5) can be higher or lower in the MutSL^+^ versus the MutSL^−^ context. These variations result from the balance between the strength of the mutator phenotype of the replication mutants (assessed from the mutant proportion in the MutSL^−^ context) and the ability of the MutSL system to correct polymerase errors (assessed from the difference in the mutant proportion in the MutSL^+^ and MutSL^−^ backgrounds).

Collectively, these results show that DnaE errors, similar to PolC misincorporations, are corrected by the mismatch repair system post-replication and hence confirm indirectly that DnaE synthesizes DNA within the replisome. More importantly, the clear mutagenesis increase in the double *polC dnaE* mutants over *polC* mutants support the notion that DnaE can replicate a significant part of the chromosome in cells encoding mutator forms of both PolC and DnaE.

### 3.6. The rate of DnaE errors *in vivo* correlates inversely with PolC concentration

The results presented above show that nucleotides polymerized by DnaE during DNA synthesis are stably incorporated in the chromosome. However, to accurately fulfil this task, DnaE fidelity should be drastically improved [35,39,45]. This is in part achieved by the mismatch repair system that removes DnaE misincorporations post-replication. However, as is the case with all other replicative polymerases, DnaE misincorporations at growing 3′-OH ends should be corrected by a proofreader. These 3′ > 5′ exonuclease proofreaders are either integral parts of the same polymerase polypeptides or separate proteins interacting with polymerases [65]. PolC contains a domain endowed with a 3′ > 5′ exonuclease activity that corrects misincorporations and its inactivation results in a strong mutator phenotype [21,22,32]. As DnaE has no 3′ > 5′ exonuclease activity [23,28,32,35] (figure 3), we hypothesized that a separate protein may provide a proofreading activity *in trans*. In order to test this hypothesis, we studied several candidates in *B. subtilis*. DinG, KapD, YprB and PpsA have 3′ > 5′ exonuclease motifs, while YhaM has 3′ > 5′ exonuclease activity on ssDNA but no homology with typical DNA polymerase proofreaders [66,67]. The corresponding genes were inactivated and the relevant strains tested for spontaneous mutagenesis using the rifampin resistance (Rif^R^) assay. The PolC exonuclease mutant *mut1A* was also tested as a positive control. While *mut1A* conferred a strong mutator phenotype, there was no increase in mutagenesis in the *dinG*, *kapD*, *yprB*, *ppsA* and *yhaM* null mutants (electronic supplementary material, figure S6), indicating that none of these proteins provide the DnaE proofreader. Therefore, DnaE misincorporations are either corrected by a new undiscovered proofreader or by the 3′ > 5′ exonuclease activity of PolC. The structural PolC–DnaE interaction detected previously by yeast two-hybrid [39] may provide the physical contact needed for proofreading of DnaE errors by the PolC 3′ > 5′ exonuclease *in trans*. Similar correction of polymerase errors *in trans* by proofreaders has been observed before in *E. coli* and yeast [68–72].

**Figure 6.**
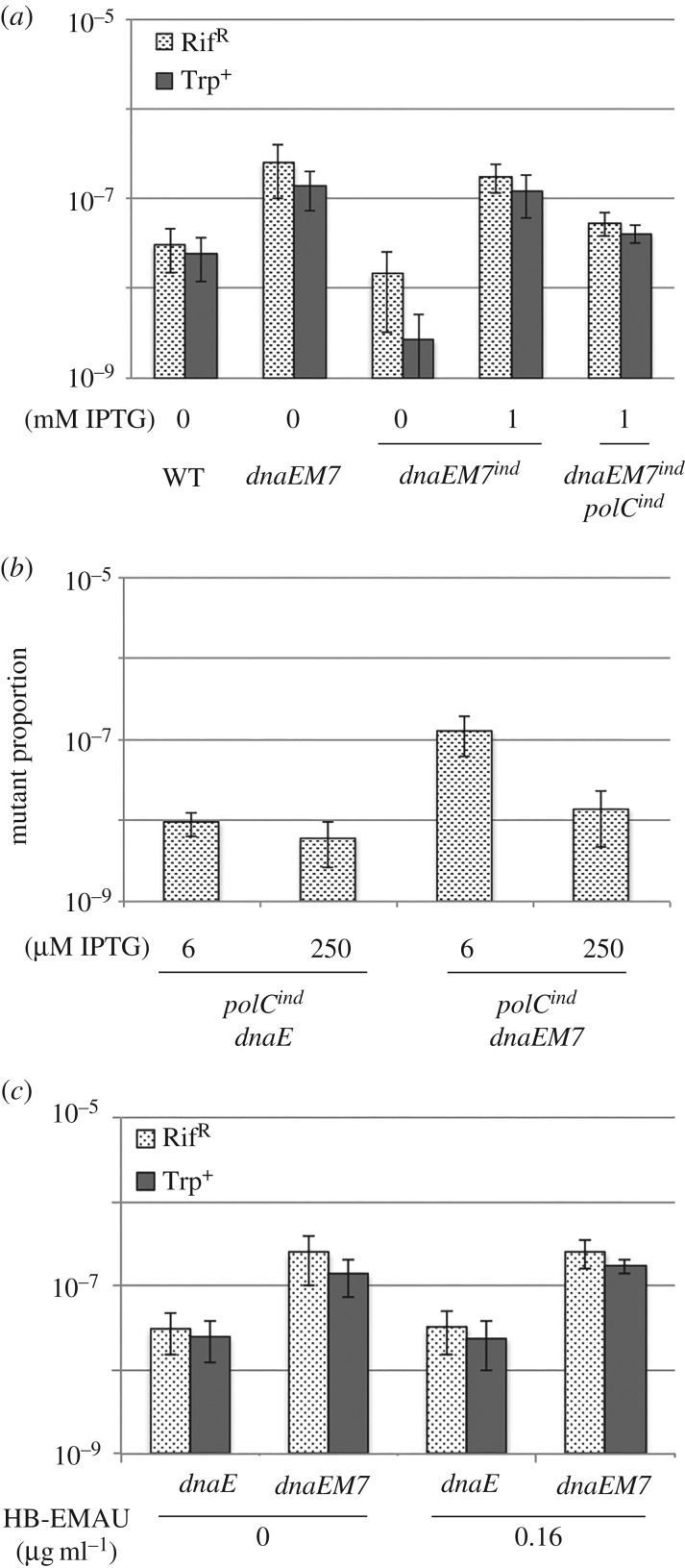
The rate of DnaE misincorporations inversely correlates with PolC concentration. (*a*) Mutagenesis in DnaEM7 inducible strains. Isolated colonies of isogenic TrpC^−^ strains encoding DnaEM7 (*dnaEM7^ind^*) or DnaEM7 and PolC (*dnaEM7^ind^ polC^ind^*) from the inducible *Pspac* promoter were obtained in plates containing 50 µM IPTG. Six independent colonies were then cultivated for about 10 generations in 0 or 1 mM IPTG and plated on 50 µM IPTG to measure the proportion of Rif^R^ and Trp^+^ cells. The mutagenesis rate of the WT and *dnaEM7* strains was also determined from six isolated colonies. WT: JJS9; *dnaEM7*: DGRM850; *dnaEM7^ind^*: DGRM838; *dnaEM7^ind^ polC^ind^*: DGRM848*.* (*b*) Effect of PolC depletion on the mutator phenotype of DnaE and DnaEM7. Strains encoding *dnaE* or *dnaEM7* from natural expression signals and *polC* from the IPTG-dependent *Pspac* promoter (*polC^ind^*) were grown in LB plates with 250 µM IPTG. Six independent colonies were then cultivated over a day to carry out about 15 generations at 6 or 250 µM IPTG (note that cell growth ceased at ≤3 µM IPTG). The proportion of Rif^R^ cells was determined from freshly saturating cultures. *polC^ind^ dnaE* (DGRM840); *polC^ind^ dnaEM7* (DGRM841). (*c*) Effect of HB-EMAU on DnaE and DnaEM7 mutagenesis. Cells encoding DnaE or DnaEM7 from natural expression signals were streaked on LB plates. Six isolated colonies were then grown in the presence or absence of 0.16 µg ml^−1^ HB-EMAU. At saturation, cells were plated to determine the proportion of Rif^R^ and Trp^+^ mutants. Similar results were obtained at 0.3 µg ml^−1^ HB-EMAU. WT: JJS9; *dnaEM7*: DGRM850.

If DnaE misincorporations are indeed corrected *in trans* by PolC *in vivo*, we hypothesized that the rate of spontaneous mutagenesis in cells encoding a mutator form of DnaE will vary with the DnaE/PolC ratio: it will increase in cells with a high ratio and decrease in cells with a low ratio. By comparison, the mutagenesis rate will be unaffected by changes in the DnaE/PolC ratio if the proofreading activity of DnaE is provided by another unidentified factor. To test the effect of the DnaE/PolC ratio on DnaE-dependent mutagenesis, we measured the proportion of Rif^R^ and Trp^+^ mutants in cells encoding DnaEM7 from the natural promoter or from the IPTG-dependent *Pspac* promoter in MutSL^+^ cells. In the IPTG-dependent strain, the intracellular amount of DnaE increases with IPTG concentrations from about 50 to 1000 molecules per cell, a range that covers the physiological intracellular amount of DnaE (approx. 300 molecules) [35]. We found that DnaEM7 depletion (0 µM IPTG) decreased the proportion of Rif^R^ and Trp^+^ mutants, whereas threefold overexpression (1 mM IPTG) of DnaE had no effect on mutagenesis compared to the parental DnaEM7 strain (figure 6*a*). When PolC and DnaEM7 were both overexpressed from the IPTG-inducible promoter (like DnaE, the PolC construction is expected to produce tens to one thousand of PolC molecules per cell, a range that covers the physiological concentration of PolC, approx. 100 molecules [27,34]), the mutagenesis rate decreased compared with cells overexpressing DnaEM7 alone. These data show that the mutagenesis rate in the *dnaEM7* background correlates proportionately with the DnaE/PolC ratio *in vivo*.

To further test the interplay between the DnaE/PolC ratio and DnaEM7 mutagenesis, the DnaE/PolC ratio was increased by depleting PolC. To do this, we used strains encoding PolC from the *Pspac* promoter and DnaE or DnaEM7 from its natural locus. The rate of spontaneous mutagenesis after 15 generations at 6 and 250 µM IPTG was monitored using the Rif^R^ assay (pre-cultures were carried out at 250 µM IPTG). The mutagenesis rate in *dnaEM7* cells was about 5–10 times higher at 6 compared with 250 µM IPTG (figure 6*b*). This increase may result from a deficiency in correction of DnaEM7 errors at low levels of PolC expression as (i) the mutagenesis rate remained at basal level at high and low IPTG concentrations in the DnaE context, and (ii) Rif^R^ bacteria did not prevail in the culture because they grew at a rate similar or even lower than Rif^S^ cells [64] (our observations).

However, as PolC is a major replicative polymerase, depleting its cellular concentration may profoundly affect DNA synthesis causing accumulation of unscheduled prematurely terminated 3′-OH ends that may then be opportunistically extended by DnaE. Hence, the stimulation of mutagenesis observed upon PolC depletion may result from an increase in the amount of DNA replicated by DnaEM7 rather than from a decrease in DnaE proofreading activity. To distinguish between these possibilities, we analysed DNA replication at 6 µM and 250 µM IPTG using a marker frequency approach. For this, we measured by qPCR the ratio of DNA at the replication origin (*ori*) versus the replication terminus (*ter*) in cells growing exponentially at 6 or 250 µM IPTG and normalized the ratio with the *ori/ter* ratio of non-replicating cells. In cells expressing DnaE and PolC from their natural locus, the *ori/ter* ratio is about 4.2 (figure 1). A physiological ratio was found at both IPTG concentrations (4.2 at 6 µM and 3.8 at 250 µM), while it can rise up to 16 when PolC is dramatically depleted (figure 1; see below). This shows that DNA replication is, at most, marginally affected at 6 µM IPTG, demonstrating that the mutagenic phenotype observed at low PolC concentration does not depend on notable replication defects.

By specifically trapping PolC at 3′-OH ends of growing chains in an inactive and stable complex [26,30], HB-EMAU allowed us to alter the DnaE/PolC ratio by poisoning the PolC polymerase activity without changing its concentration and without producing free extra 3′-OH ends. The mutagenesis in cells expressing DnaE or DnaEM7 from the wild-type locus was thus analysed in the presence of the highest HB-EMAU concentrations (0.16 and 0.32 µg ml^−1^) compatible with growth and 100% survival in the absence of the RecA protein (electronic supplementary material, figure S7*a*,*b*). At these concentrations, DNA elongation was impeded, causing an increase in the *ori/ter* ratio from approximately 4 (the basal level) to 7.1 and 10.1, respectively (electronic supplementary material, figure S7*c*). Under these conditions of clearly compromised PolC polymerase activity, the Rif^R^ and Trp^+^ mutagenesis assays did not show any change in the mutagenesis rate in DnaE and DnaEM7 cells (figure 6*c*). This suggests that DnaEM7 mutagenesis observed in PolC-depleted cells results from a reduction in PolC polymerase concentration rather than from a decrease in PolC activity, accumulation of free 3′-OH ends and/or an abnormally high amount of DNA replicated by DnaE.

Collectively, our data show that DnaEM7-dependent mutagenesis varies inversely with PolC concentration (and not with PolC polymerase activity), implying that a key cellular factor controlling DnaE misincorporations *in vivo* is the 3′ > 5′ exonuclease activity of PolC. These results suggest that the high rate of mutagenesis observed in the PolC exonuclease mutant *mut1A* results from a reduction in proofreading of both PolC and DnaE errors rather than of PolC alone.

### 3.7. DnaE recruits PolC at primed sites and exposes the 3′-OH primer end to the 3′ > 5′ exonuclease activity of PolC

**Figure 7.**
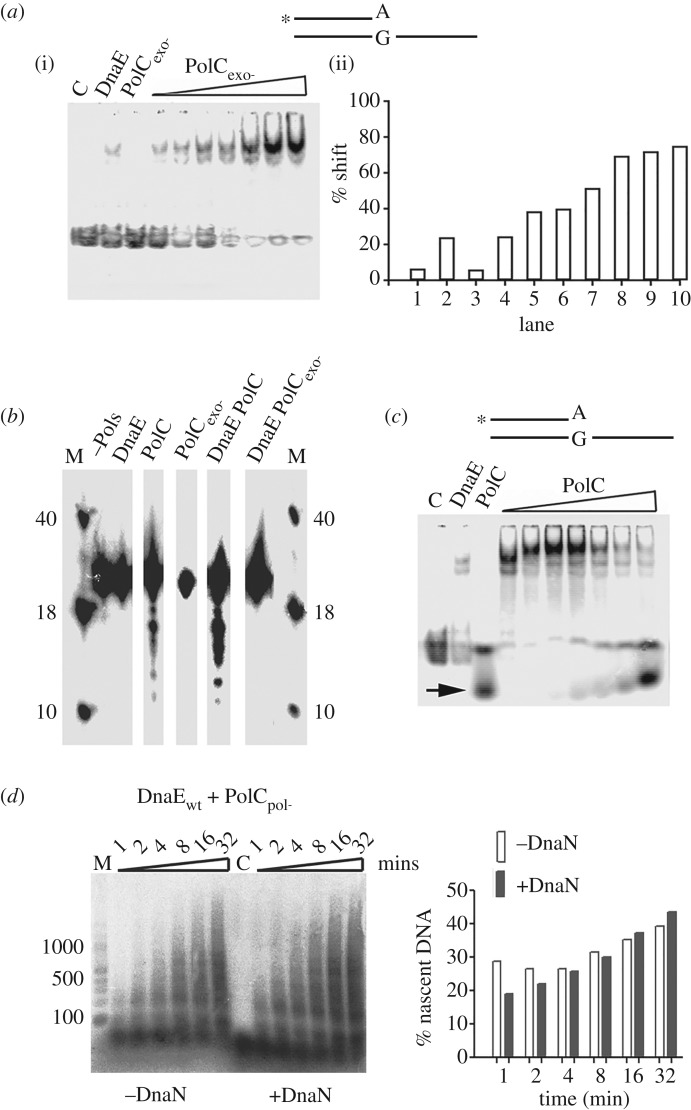
PolC is recruited by DnaE at 3′-OH primed sites via protein–protein interaction and DnaE exposes 3′-OH ends to the exonuclease domain of PolC. (*a*) EMSA showing the effects of increasing concentrations (25, 50, 100, 250, 500, 750 and 1000 nM) of PolC_exo-_ on complexes of DnaE (1 µM) with radiolabelled (asterisk) primed 3′-mismatch (A:G) DNA substrate (0.66 nM). Lanes labelled C, DnaE or PolC_exo-_ represent the control radiolabelled DNA substrate on its own, in the presence of 1000 nM DnaE, or PolC_exo-_, respectively. Quantification of the percentage shift in every lane is shown in the bar graph. (*b*) PolC exonuclease assays. The assays were carried out with 80 nM of DnaE, PolC or PolC_exo-_ or with equimolar concentrations of DnaE and PolC (wild-type and mutants) proteins. Reaction mixtures were pre-incubated at 37°C for 5 min prior to initiation of the reaction by adding the appropriate polymerase(s). The reactions were terminated after 15 min by boiling for 5 min. Samples were mixed with loading buffer and resolved through 15% (v/v) urea gel denaturing polyacrylamide gel. Lanes labelled M show molecular markers (40, 18 and 10 bases) and the lane labelled –Pols represents the control radiolabelled substrate. (*c*) EMSA showing the effects of increasing concentrations (25, 50, 100, 250, 500, 750 and 1000 nM) of wild-type PolC on complexes of DnaE (1 µM) with radiolabelled (asterisk) primed 3′-mismatch (A:G) DNA substrate (0.66 nM). Lanes labelled C, DnaE or PolC represent the control radiolabelled DNA substrate on its own, in the presence of 1000 nM DnaE, or PolC, respectively. DNA fragments digested by the 3′ > 5′ exonuclease activity of PolC are shown with an arrow. (*d*) Time course reactions carried out on RNA-primed M13mp18 template (2 nM) with DnaE and PolC_pol-_ (80 nM each) and in the presence or absence of DnaN (80 nM) as indicated. Reactions were carried out and analysed as in figure 4. Quantifications of the percentage of nascent DNA synthesized in the presence and absence of DnaN are shown in the bar graph.

In order to directly test the hypothesis of *trans* proofreading, we investigated whether DnaE bound to a primed site can physically recruit PolC and expose the 3′-OH end for degradation by the 3′ > 5′ exonuclease activity of PolC. We first carried out an EMSA assay to look for a ternary template–DnaE–PolC complex. Because we were using a synthetic DNA-primed substrate, this study was carried out with a PolC protein inactivated for the 3′ > 5′ exonuclease activity and thus unable to degrade DNA primers (PolC_exo-_) (electronic supplementary material, figure S8—compare panels A and B with panel E). EMSA controls showed that PolC_exo-_ on its own is unable to form a detectable stable complex with fully paired DNA-primed substrates (electronic supplementary material, figure S9, right panel) as well as with mispaired (A:G) 3′-OH end template (figure 7*a*(i), lane PolC_exo-_). In contrast, DnaE forms stable complexes with these two substrates (figure 7*a*(i), lane DnaE; electronic supplementary material, figure S9, left panel). In the EMSA assays with the mismatched template pre-incubated with DnaE and then increasing concentrations of PolC_exo-_, a stable ternary complex formed (figure 7*a*(i)). The percentage of shifted substrate increased gradually along with PolC_exo-_ concentration up to fourfold the signal with DnaE alone (figure 7*a*(ii)). This shows that, in full reaction mixtures, PolC_exo-_ is recruited to the mispaired template by DnaE to form a stable ternary template–DnaE–PolC_exo-_ complex.

To determine whether DnaE protects or exposes bound 3′-OH ends of DNA chains to the 3′ > 5′ exonuclease activity of PolC in the ternary template–DnaE–PolC complex, we carried out 3′ > 5′ exonuclease assays using a DNA primer annealed onto ssM13mp18. Results showed that DNA primers were digested by PolC but not PolC_exo-_ and that the degradation was more potent in the presence than in the absence of DnaE (figure 7*b*). Furthermore, we carried out EMSA with the mispaired (A:G) 3′-OH end synthetic template pre-incubated with DnaE and then adding increasing concentrations of wild-type PolC. A strong ternary complex formed initially followed by digestion of the DNA by the 3′ > 5′ exonuclease activity of wild-type PolC (figure 7*c*).

The combined data from EMSA and the exonuclease assays showed that DnaE binds to primed 3′-OH DNA ends, recruits PolC via a physical interaction forming a stable template–DnaE–PolC ternary complex and exposes the 3′-OH end of the primer to the 3′ > 5′ exonuclease activity of PolC for proofreading *in trans*. The fact that DnaE and PolC form a stable ternary complex at paired and mispaired 3′-OH ends suggests that PolC interacts with DnaE while DnaE synthesizes Okazaki fragments, opening the possibility of instantaneous PolC-mediated proofreading of DnaE errors *in trans*.

### 3.8. The proofreading domain of PolC stimulates DnaE-dependent synthesis *in vitro*

To obtain further evidence of PolC-mediated proofreading of DnaE misincorporations during DnaE-dependent DNA synthesis, primer extension assays were carried out. Assuming that DnaE errors slow down nascent strand synthesis [39], we argued that if the 3′ > 5′ exonuclease activity of PolC were to proofread DnaE errors within a stable ternary template–DnaE–PolC complex, then DnaE-mediated nascent DNA synthesis will be improved in DnaE/PolC mixtures compared with reactions carried out with DnaE alone. However, if another distinct factor were to proofread DnaE errors then the presence of the 3′ > 5′ exonuclease activity of PolC will not change the fidelity of DnaE and similar replication products (in size and yields) will be generated by the two reaction mixtures. This analysis was carried out using primer extension assays containing an RNA-primed ssM13mp18 template, DnaE and an exonuclease proficient, polymerase-deficient PolC protein (PolC_pol-_). Control experiments on short primed oligonucleotides showed that the PolC_pol-_ protein has an active exonuclease domain but no polymerase activity (electronic supplementary material, figure S8*d*), compared with PolC that extends and degrades DNA- but not RNA primers, as shown previously (electronic supplementary material, figure S8*a*–*c*) [23,27,29].

Primer extension assays with RNA-primed ssM13mp18 template revealed stimulation of DnaE-dependent DNA synthesis in the presence of the PolC 3′ > 5′ exonuclease (compare the left parts of figures 4*b* and 7*d*). Although results presented in figure 7*a*,*c* showed that DnaE binds the primed site and recruits PolC to form a stable ternary complex, part of the nascent strands shown in figure 7*d* may result from occasional dissociation of the ternary complex followed by excision of mispaired 3′-OH ends through a transient interaction with the 3′ > 5′ exonuclease of PolC_pol-_ (electronic supplementary material, figure S8*d*) and reloading of the ternary complex. To eliminate this possibility, we carried out primer extension assays in the presence of DnaN, a protein that tethers DNA polymerases on primed templates and prevents their dissociation from growing chains [3,5]. The DnaE–DnaN interaction was previously found to strongly improve the processivity of DnaE (greater than 7 kb) and to stimulate its polymerase activity [23,29]. Results showed that the stimulation of DnaE polymerase activity by PolC_pol-_ is slightly improved rather than inhibited in reactions containing DnaN (figure 7*d*; compare the quantifications of the gel in the bar graph). Overall, our data reveal a functional interaction between the DnaE polymerase and PolC 3′ > 5′ exonuclease activities that improves DnaE-mediated DNA synthesis, and further suggest that DnaE misincorporations are corrected *in trans* by the PolC 3′ > 5′ exonuclease activity *in vitro*, consistent with our *in vivo* data (figure 6).

### 3.9. DnaN inhibits the error-prone activity of DnaE

Another important process during accurate replication of DNA is the selection of the correct nucleotide at the catalytic site of polymerases and the inhibition of extension at mispaired 3′-OH ends [43]. Previous studies showed that DnaE is error-prone as a result of a promiscuous active site permitting high frequency of base misinsertions and extensions of mispaired 3′-OH ends on damaged and intact templates [35,39,45]. In primer extension assays with intact DNA templates carried out in the absence of dATP and dGTP, we previously found that DnaE efficiently bypasses at T and C positions along the template and this error-prone synthesis is reduced by interactions with DnaG and DnaC [39].

**Figure 8.**
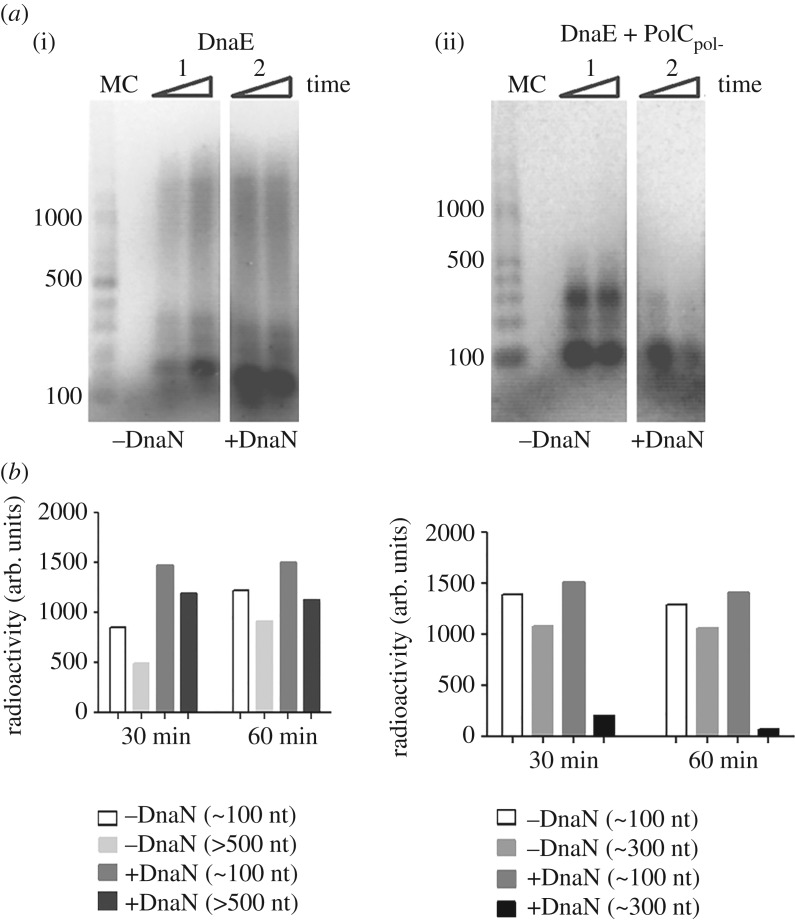
Effect of DnaN and/or PolC_pol-_ on DnaE error-prone polymerase activity. Primer extension assays were carried out on RNA primed ssM13mp18 DNA omitting dGTP. Time course reactions at 30 and 60 min (indicated by rectangular triangles) were terminated by addition of loading dye and incubation at 95°C for 5 min. Primer extension products were analysed through 1.5% (w/v) alkaline agarose gels. (*a*) DnaE (360 nM) and (*b*) DnaE and PolC_pol-_ at equimolar concentrations (360 nM) in the presence or absence of DnaN (360 nM), as indicated. Lanes labelled M and C show molecular weight markers and a control without polymerases, respectively. All assays were carried out in triplicates and the gels shown here are representative. Quantifications of nascent DNA synthesized (radioactivity measured in arbitrary units, AU) are shown in bar graphs underneath the gels.

In order to search for additional protein–protein interactions that reduce the error-prone polymerase activity of DnaE, we analysed the effect of DnaN and PolC_pol-_ on DnaE fidelity. We used ssM13mp18 templates primed with a radioactively labelled RNA with dGTP omitted from all the replication reactions, forcing DnaE errors at cytosines along the template (see Material and methods for details). Results showed that DnaE alone was able to synthesize substantial amounts of long (greater than 500 nt) nascent DNA fragments (figure 8*a*(i), lane 1). In the presence of DnaN, a strong accumulation of small (approx. 100 nt) fragments was observed in addition to long (greater than 500 nt) fragments (figure 8*a*(i), compare lanes 1 and 2 and also the quantification bar graph shown below the gel). We hypothesized that long fragments were products of DnaE alone while small fragments were products of DnaE clamped on the template by DnaN. In reactions containing DnaE and PolC_pol-_, an accumulation of small (approx. 100 nt, the major product) and moderate (approx. 300 nt) size fragments was observed (figure 8*a*(ii), lane 1 and also the quantification bar graph shown below the gel). In the presence of DnaE, PolC_pol-_ and DnaN, only small (approx. 100 nt) DNA fragments were produced (figure 8*a*(ii), lane 2 and also the quantification bar graph shown below the gel).

Collectively, these data confirm the strong error-prone polymerase activity of DnaE and show that this activity is inhibited when DnaE is clamped at extending 3′-OH ends by DnaN. The accumulation of small (approx. 100 nt) DNA fragments in the presence of PolC_pol-_ may result from a slow down of DnaE-dependent synthesis because of *trans* proofreading of DnaE misincorporations at C residues by the PolC 3′ > 5′ exonuclease activity. The synthesis of longer fragments (approx. 300 nt) may indicate that the DnaE–PolC_pol-_ interaction has no effect on the DnaE error-prone activity or that some DNA synthesis is carried out by DnaE alone. We hypothesise that DnaN, in a similar manner to DnaG and DnaC, improves the fidelity of DnaE. These and previous data [39] show that at least three (DnaN, DnaC and DnaG) out of the six interactants of DnaE improve its fidelity by reducing its error-prone activity during both initiation and elongation of Okazaki fragment synthesis.

## 4. Discussion

Bacterial DNA replication machineries are diverse, as exemplified by the use of twin DNA polymerases in *E. coli* and two distinct enzymes in *B. subtilis*, PolC and DnaE. Several *in vivo* and *in vitro* data in *B. subtilis* and related Gram-positive bacteria showed that PolC plays a major role in chromosome replication, while DnaE is mainly involved in lagging strand synthesis (see Introduction for details). Furthermore, the promiscuous catalytic site of DnaE, endowing the enzyme with a strong error-prone activity, and the absence of any detectable intrinsic 3′ > 5′ proofreading activity, are incompatible with the idea that DnaE has a substantial role in chromosome replication in these microorganisms, as this would result in mutagenesis and compromise genome fidelity.

The current model of *B. subtilis* replication, based on an *in vitro* study involving 13 enzymes and a double-stranded, rolling-circle DNA template, suggests that PolC is the main polymerase for both the leading and lagging strand synthesis, while DnaE plays a minor role by briefly extending small RNA primers synthesized by DnaG before handing off DNA synthesis to PolC [29,39]. This model is consistent with the poor fidelity of DnaE [35,39,45], its ability to extend RNA primers whereas PolC is unable to do so (figure 3*c*; electronic supplementary material, figure S8C) [29,35], with the rather slow DnaE speed reported here and in previous work in the presence or absence of DnaN (figure 4) [23,29,35], and with the dynamics of PolC which is constantly recruited to and released from the replisome *in vivo* [34]. Moreover, the limited DnaE-dependent synthesis downstream of RNA primers may provide an elegant solution for removing the inaccurate DnaE replication products during termination of Okazaki fragment synthesis.

Interestingly, we and others found that DnaE alone is able to produce rather long nascent DNA and that this activity is significantly stimulated by DnaN, SSB and PolC (figures 4*b*,*c* and 7*d*) [23,35]. Moreover, the stimulated polymerase activity of DnaE may be functionally significant within the replisome as (i) DnaE bound to primed templates recruits PolC to form a ternary complex in which DnaE errors are proofread by the 3′ > 5′ exonuclease of PolC (figure 7), and (ii) interactions of DnaE with DnaN, SSB, the primase DnaG, the helicase DnaC, and the HolA subunit of the clamp loader may couple loading of DnaN and DnaE on RNA-primed, SSB-coated templates [36,37,39,40]. Consistently, we found previously that DnaE, DnaG and DnaC form a ternary complex in which DnaE receives very small di-ribonucleotide primers from DnaG through direct hand-off [39]. Although minimal systems do not constitute actual representation of the complete *in vivo* replisome, the data presented here suggest that DnaE can, should it be required, assume a more prominent role in lagging strand synthesis than proposed by the current model. This notion is supported in cells compromised in PolC activity by the clear increase in the mutagenesis rate of cells encoding mutator forms of both *polC* and *dnaE* compared with *polC* mutants (figure 5). Revealing here a more prominent role for DnaE in DNA synthesis invites questions about the full extent of catalytic roles of DnaE within the *B. subtilis* replisome. Moreover, the DnaE network of protein interactions has no equivalent in *E. coli*, indicating that the mechanisms of lagging strand synthesis in *E. coli* and *B. subtilis* are significantly different.

Polymerase asymmetry is found in eukaryotes with three replicative polymerases (Polα, Polδ and Polε) working cooperatively to provide an elegant solution to the problem of asymmetric DNA strand synthesis [3]. Polα synthesizes *de novo* short RNA primers with its primase activity and extends them with its DNA polymerase activity for about 20 nt. Several *in vivo* and *in vitro* independent studies showed that Polε and Polδ take over from Polα on the leading and lagging strands, respectively, to synthesize the bulk of nascent DNA [73–77].

Bacterial replicative DNA polymerases are high-fidelity enzymes that incorporate and extend mispaired nucleotides at a very low frequency [43]. A striking exception to this rule is the DnaE2 group of C family DNA polymerases that are non-essential enzymes associated with translesion synthesis (TLS) in DNA-damage tolerance and in induced mutagenesis [14,15]. However, the replicative DnaE polymerase of *B. subtilis* and related bacteria, which belongs to the DnaE3 type of C family DNA polymerases, is also endowed with an error-prone activity: in *in vitro* polymerase assays it incorporates and extends mispaired nucleotides at a very high frequency in damaged and native templates [35,39,45] (figure 8). However, unlike Y-type error-prone polymerases, DnaE overproduction *in vivo* does not increase the rate of spontaneous mutagenesis [35,46,47]. Moreover, although DnaE depletion prevents UV-induced mutagenesis in *B. subtilis* [35], mutagenesis at UV damage sites is carried out by the error-prone PolY2 enzyme of the Y family of DNA polymerases (assisted by DNA Pol I) rather than by DnaE [47,78]. Clearly, DnaE errors are swiftly prevented and/or corrected *in vivo*.

The collective results presented here show that (i) DnaE interactions with DnaN strongly reduce the error-prone activity of DnaE (figure 8) (interactions with DnaC and/or DnaG also reduce this activity [39]), (ii) DnaE misincorporations are proofread by the 3′ > 5′ exonuclease activity of PolC *in trans* via a stable template–DnaE–PolC ternary complex (figures 6 and 7), and (iii) the mismatch repair system removes DnaE misincorporations at the replication fork (figure 5). This reaction may involve the interaction of DnaE with MutS and MutL [37]. Hence, the cumulative evidence suggests that a multitude of DnaE interactions with replisomal proteins improves the fidelity of DnaE at the insertion/extension and also at the proofreading steps during replication, as well as post-replication by the mismatch repair system, as observed for all the bona fide replicative polymerases known so far. The discovery of factors that strongly improve the fidelity of DnaE supports the hypothesis that DnaE can accurately replicate substantial amounts of the *B. subtilis* genome if needed, for example when PolC activity is somehow compromised.

In eukaryotes, Polα initiates Okazaki fragment synthesis on average every 165 nt by synthesizing a 30–35 nt primer with RNA at the 5′ end and DNA at the 3′ end. This polymerase thus contributes significantly to the total amount of DNA synthesis during replication [79]. This significant contribution to replication, coupled with the enzyme's lack of proofreading activity, necessitates compensation by Polδ and the mismatch repair system to proofread *in trans* and correct errors created by Polα [71,80], analogous to what we propose here for DnaE and the 3′ > 5′ exonuclease activity of PolC in *B. subtilis*.

The observations that DnaE belongs to the SOS regulon, is required at high concentration for UV-induced mutagenesis and endowed with an error-prone activity in the absence of auxiliary replisomal proteins [35,39,45,81] suggest that DnaE may also be acting as an alternative polymerase for DNA repair and possibly TLS. TLS is an extremely important mechanism that supports DNA synthesis over lesions that could otherwise not be handled by the high-fidelity replicative DNA polymerases. For this to happen, DnaE will probably need to dissociate temporarily from the replisomal proteins DnaC, DnaG, PolC and/or DnaN that improve its fidelity within the replication fork. Once it has passed (or helped DNA synthesis) through the lesion, DnaE can re-associate with the replisome to resume accurate replication. Additional investigations are awaited to better understand the complex involvement of DnaE in DNA replication and repair.

Our *in vivo* data also show that DnaE is involved in the initiation of replication and that this polymerase is functionally loaded at *oriC* before PolC where it performs mandatory synthesis prior to PolC-dependent elongation (figure 1; electronic supplementary material, figure S4). This is consistent with data showing that DnaE interacts with DnaC and DnaG [39], the first two proteins loaded at *oriC* by DnaA during initiation [1], and with results showing that DnaE, but not PolC, extends RNA primers synthesized by DnaG (figure 3*c*; electronic supplementary material, figure S8C) [29,35,39]. While in *E. coli* loading of the replicative helicase, the primase and subsequent synthesis of the first RNA primers within *oriC* signal the completion of the primosome assembly and the end of the initiation stage of DNA replication, in *B. subtilis* the DnaE-dependent extension of the short RNA primers by a direct hand-off mechanism from the primase [39] suggests that the completion of the initiation stage of replication occurs after an initial DNA synthesis by DnaE. Although DnaE is not considered as a *de facto* primosomal protein, its activity seems to be essential for initiation of DNA replication in *B. subtilis*.

*Escherichia coli* replication is the bacterial paradigm and its replisome has provided the main structural and mechanistic model in the field. However, the peculiarities of the *E. coli oriC* and its replication proteins suggest that it may not be a good representative universal bacterial model [1,6,7]. It is increasingly becoming apparent that bacterial replication systems vary considerably. This divergence is obvious even at the level of DNA synthesis that involves only one replicative polymerase in *E. coli* and two (PolC and DnaE) in *B. subtilis*. PolC/DnaE-containing bacteria, like *B. subtilis*, diverged from *E. coli* three billion years ago [82], and their replisomes are distinct in terms of composition and structural organization [6,7,39].

## Supplementary Material

ESM file

## Data accessibility

Data supporting this article have been uploaded as part of the electronic supplementary material.
