# Correction to ‘Interactions of the *Bacillus subtilis* DnaE polymerase with replisomal proteins modulate its activity and fidelity’

**DOI:** 10.1098/rsob.170234

**Published:** 2017-11-08

**Authors:** Vasileios Paschalis, Emmanuelle Le Chatelier, Matthew Green, Hamid Nouri, François Képès, Panos Soultanas, Laurent Janniere

*Open Biol.* **7**, 170146. (Published online 6 September 2017). (doi:10.1098/rsob.170146).

## Correction details

A correction is required to the author list and contributions statements for ‘Interactions of the *Bacillus subtilis* DnaE polymerase with replisomal proteins modulate its activity and fidelity’ doi:10.1098/rsob.170146. All the authors gave their consent for the addition of the author, Dr Hamid Nouri. The updated list is as follows:

## Authors

Vasileios Paschalis^1,†^, Emmanuelle Le Chatelier^2,†,‡^, Matthew Green^1,†^, Hamid Nouri^3,^^¶^, François Képès^3,||^, Panos Soultanas^1^, Laurent Janniere^3,||^
^1^Centre for Biomolecular Sciences, School of Chemistry, University Park, University of Nottingham, Nottingham NG7 2RD, UK
^2^Institut National de la Recherche Agronomique, Génétique Microbienne, 78350 Jouy-en-Josas, France
^3^iSSB, Genopole, CNRS, Univ EVRY, Université Paris-Saclay, Génopole Campus 1, Genavenir 6, 5 rue Henri Desbruères, 91030 Evry, France
^†^These authors contributed equally to this study.
^‡^Present address: Institut National de la Recherche Agronomique, Metagenopolis (US1367), 78350 Jouy-en-Josas, France.
^¶^Present address: Laboratoire de Microbiologie Appliquée, Faculté des Sciences de la Nature et de la Vie, Université de Bejaia, 06000 Béjaia, Algeria.
^||^Present address: CEA, CNRS, Univ EVRY, Université Paris-Saclay, Génomique Métabolique (UMR8030), Laboratoire iSSB, Génopole Campus 1, Genavenir 6, 5 rue Henri Desbruères, 91030 Evry, France.

## Corrected Authors' contributions

E.L.C., L.J. and P.S. designed the study. V.P. and M.G. performed *in vitro* experiments. E.L.C., H.N. and L.J. performed *in vivo* experiments. All authors analysed and discussed the data. P.S. and L.J. wrote the manuscript. All the authors read and approved the final manuscript.

