# Supplementary material for: Interactions of the Bacillus subtilis DnaE polymerase with replisomal proteins modulate its activity and fidelity
Source: Open Biol. 2017 Sep 6;7(9):170146. doi: 10.1098/rsob.170146 (PMC5627055; doi:10.1098/rsob.170146)
Supplement: ESM file [file rsob170146supp1.pdf]

Interactions of the *Bacillus subtilis* DnaE polymerase with replisomal proteins modulate its activity and fidelity

Vasileios Paschalis[1,#], Emmanuelle Le Chatelier[2,§,#], Matthew Green[1,#], François Képès[3,¶], Panos Soultanas[1,*] and Laurent Janniere[3,¶,*]

[1]Centre for Biomolecular Sciences,
School of Chemistry,
University Park,
University of Nottingham,
Nottingham NG7 2RD, UK

[2]Institut National de la Recherche Agronomique
Génétique Microbienne,
F-78350 Jouy-en-Josas, France

[3]iSSB, Genopole, CNRS, Univ EVRY, Université Paris-Saclay,
Génopole Campus 1, Genavenir 6,
5 rue Henri Desbruères
F-91030 Evry, France

[§]Present address: Institut National de la Recherche Agronomique,
Metagenopolis (US1367)
F-78350 Jouy-en-Josas France

[¶]Present address: CEA, CNRS, Univ EVRY, Université Paris-Saclay,
Génomique Métabolique (UMR8030),
Laboratoire iSSB,
Génopole Campus 1, Genavenir 6,
5 rue Henri Desbruères
F-91030 Evry, France

# These authors contributed equally to this work
*Joint corresponding authors
L. Janniere: laurent.janniere@issb.genopole.fr
P. Soultanas: Panos.Soultanas@nottingham.ac.uk
DOI: 10.1098/rsob.20160146

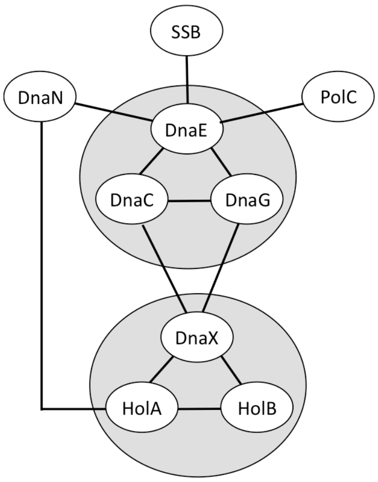

Supplemental Figure S1

Relevant protein-protein interactions in the replisome.

Small and large grey circles represent proteins and protein complexes, respectively. DnaC: helicase; DnaG: primase; DnaE: DNA polymerase; PolC: DNA polymerase; DnaX, HolA and HolB: subunits of the clamp loader; DnaN: clamp; SSB: single stranded binding protein. The DnaE-DnaC-DnaG complex is at the heart of the lagging strand half of the replisome (see the text for more details).

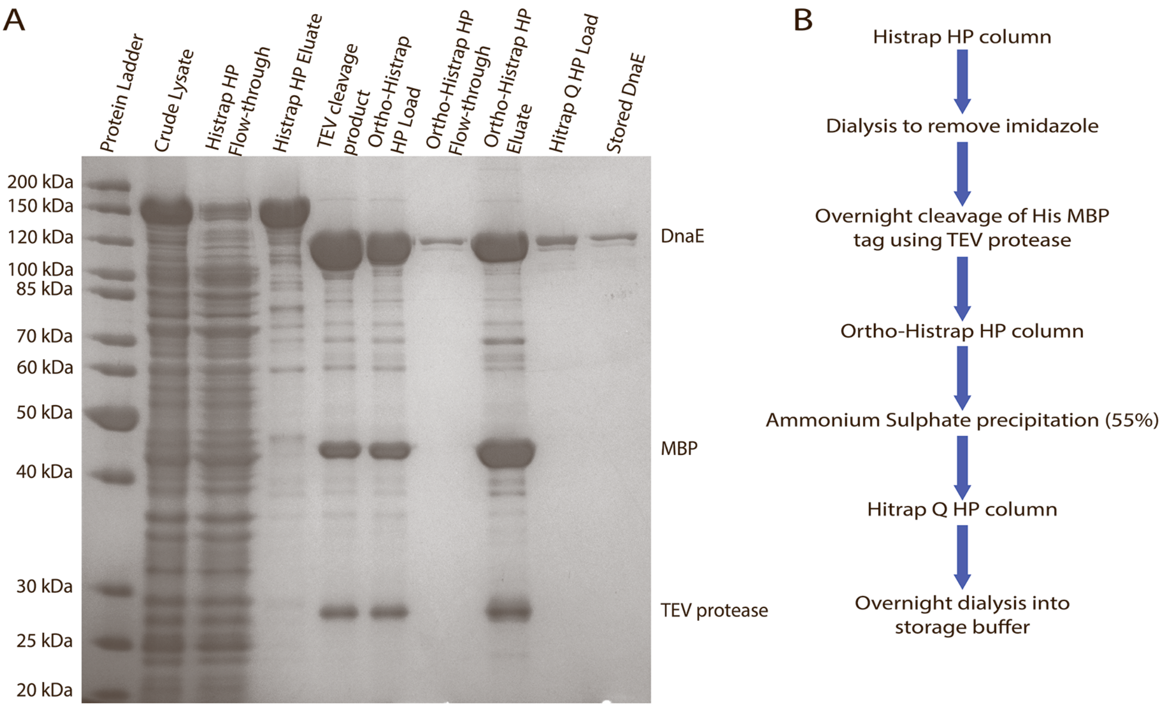

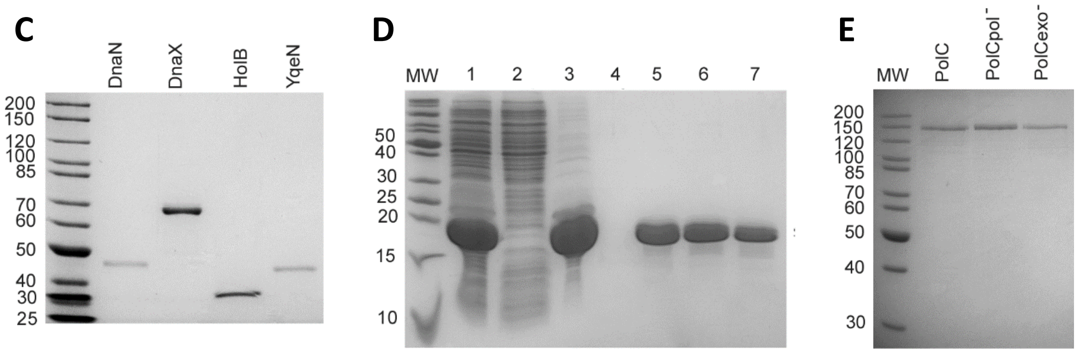

Supplemental Figure S2

SDS-PAGE gels showing the purified proteins

N-terminal hexahistidine-tag maltose binding protein cleaved DnaE purification steps (**A**) and scheme (**B**). **C**: SDS-PAGE gels showing the purified DnaN, DnaX, HolB and YqeN proteins. **D.** SDS PAGE monitoring the purification of SSB. Numbered lanes show the crude lysate (lane 1), ammonium sulphate supernatant (lane 2), the protein after the ammonium sulphate cut as was loaded onto hiTrap Q HP (lane 3), the flowthrough from the HisTrap Q HP (lane 4), the protein eluted from HisTrap Q HP (lane 5) and two fractions after the final gel filtration through a HiLoad 26/60 Superdex (lanes 6, 7). (**E**) SDS-PAGE gels showing the purified PolC, PolC$_{pol-}$ and PolC$_{exo-}$.

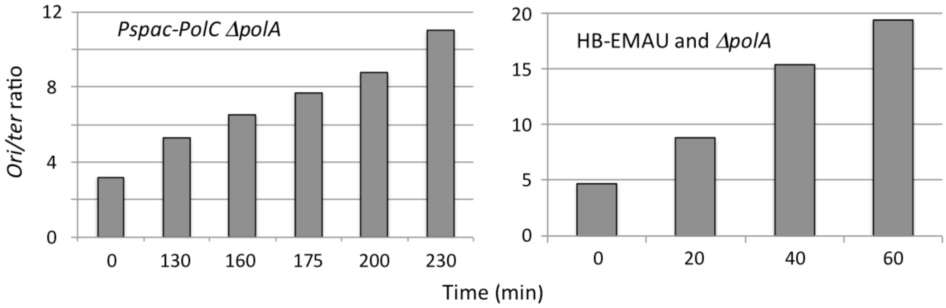

Supplemental Figure S3

The over-replication of the *oriC* region in cells depleted in PolC activity is independent on Pol I.

Left panel**:** The DGRM818 strain encoding PolC from the IPTG inducible promoter *Pspac* and deleted for *polA*, was grown in the presence of 1mM IPTG and then diluted in the absence of the inducer. The *ori/ter* ratio was determined by qPCR at different time points upon inducer removal. Right panels: Exponentially growing EDV97 cells lacking *polA* were treated with a lethal concentration of a HB-EMAU (10 µM). The *ori/ter* ratio was determined by qPCR at different time points upon HB-EMAU treatment. Representative experiments are shown.

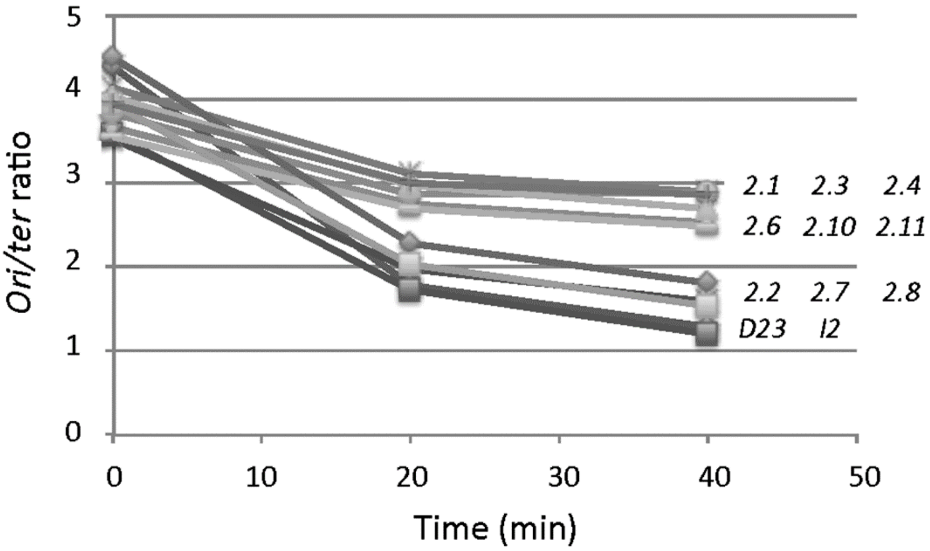

Supplemental Figure S4

Replication arrest in thermosensitive DnaE mutants.

Exponentially growing cultures of *dnaE*, *dnaD* and *dnaI* thermosensitive mutants were shifted from permissive (30°C) to restrictive (49°C) temperature and the *ori/ter* ratio was measured by qPCR before (0 min) and after (20 and 40 min) the temperature shit up. Representative experiments are shown. *D23*: *dnaD23* (L1434); *I2*: *dnaI2* (L1439); *2.1*: *dnaE2.1* (DGRM630); *2.2*: *dnaE2.2* (DGRM1); *2.3*: *dnaE2.3* (DGRM631); *2.4*: *dnaE2.4* (DGRM2); *2.5*: *dnaE2.5* (DGRM632); *2.6*: *dnaE2.6* (DGRM3); *2.7*: *dnaE2.7* (DGRM633); *2.8*: *dnaE2.8* (DGRM634); *2.10*: *dnaE2.10* (DGRM4); *2.11*: *dnaE2.11* (DGRM635).

```
                            382/4
                              ↓ ↓
BsuDnaE PIKHHLLFERFLNPERVSMPDIDIDFPDTRRDEVIQYVQQKYGAMHVAQI  411
        |::..||||||||||||||||.|:||...:||:||::|...||...|:||
EcDnaE  PLEFDLLFERFLNPERVSMPDFDVDFCMEKRDQVIEHVADMYGRDAVSQI  430
                              ↑ ↑
                            401/3
```

Supplemental Figure S5

Alignment of *E. coli* and *B. subtilis* DnaE.

The essential aspartic acid residues for the polymerase activity are located at the positions 382 and 384 for *B. subtilis* DnaE. The alignment was created using EMBOSS Water from EMBL-EBI. The full-length protein has 37.8% identity, 56.8% similarity, 6.7% gaps with an alignment score of 1922.5. The region in question showed good alignment.

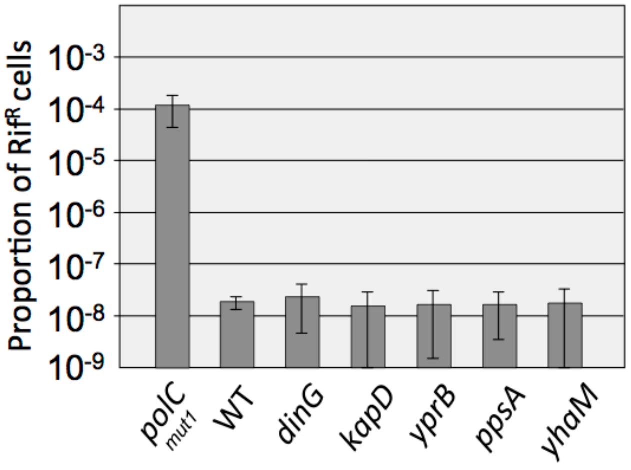

Supplemental Figure S6

Search for the DnaE proofreader

Strains encoding a WT or a mutated form of the PolC 3'>5' exonuclease proofreader ($PolC_{mut1}$) or lacking enzymes containing a domain homologous (DinG and KapD) (DGRM803-804) or distantly related (YprB and PpsA) (DGRM806, DGRM808) to proofreaders or lacking a gene endowed with a 3'>5' exonuclease activity (YhaM) (DGRM810) were tested for spontaneous mutagenesis using the Rif^R assay. Bars represent mean values and standard errors from at least six independent cultures.

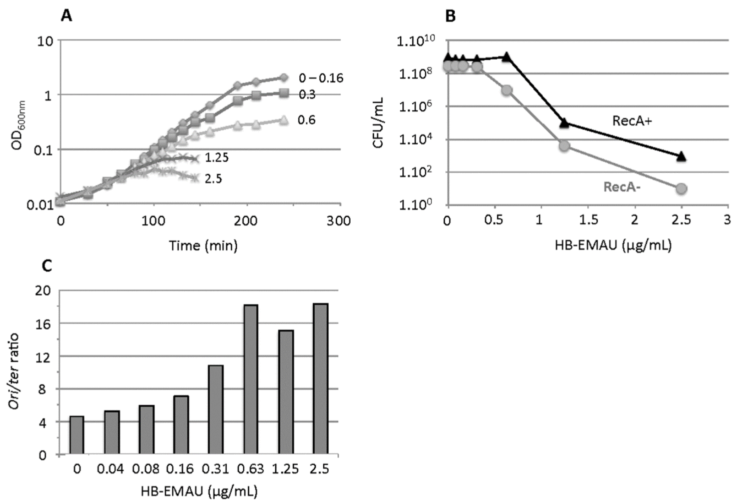

Supplemental Figure S7

Effect of HB-EMAU-induced depletion of PolC activity on growth and replication.

A. Optical analysis of the *B. subtilis* 168 strain growth in LB broth supplemented with different concentrations of HB-EMAU (µg/mL).

B: Plating efficiency of the *B. subtilis* 168 strain and its $RecA^-$ derivative (HVS567) on LB plates supplemented with different concentrations of HB-EMAU.

C: *Ori/ter* ratio at various HB-EMAU concentrations. Exponentially growing *B. subtilis* 168 cells were treated 90 min with different HB-EMAU concentrations. At OD_{600nm} of about 0.2, the total DNA was extracted and analyzed by qPCR to measure the *ori/ter* ratio. A representative experiment is shown.

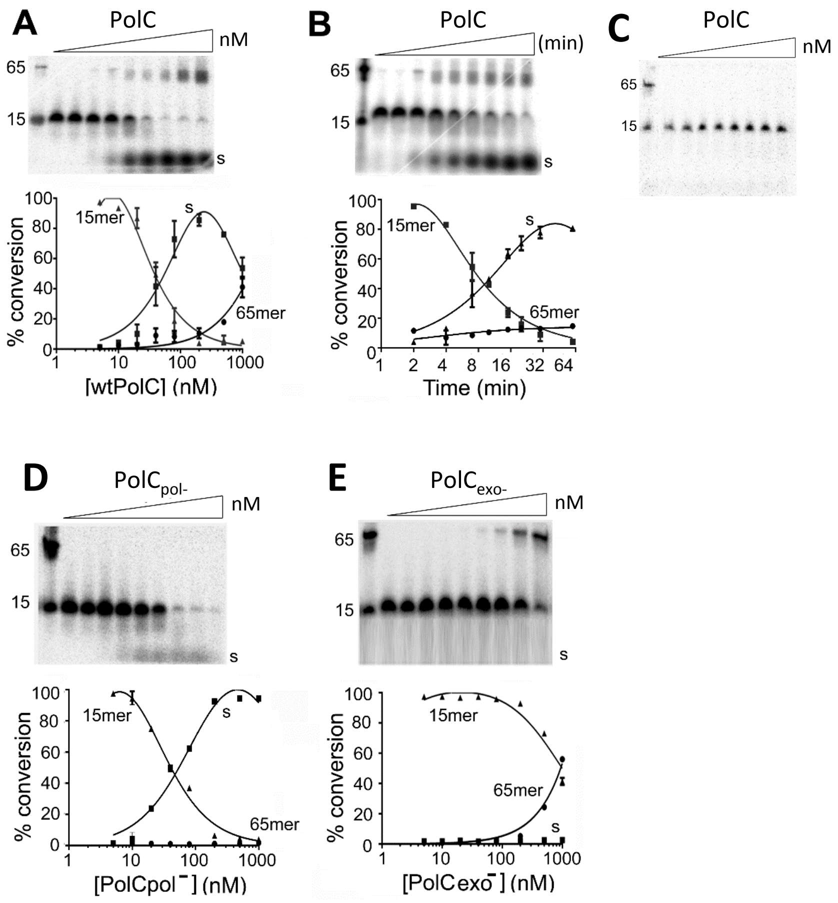

Supplemental Figure S8

B. subtilis PolC/PolC$_{pol-}$/PolC$_{exo-}$ activity assays.

Primer extension assays using short radiolabelled 15mer annealed onto longer oligonucleotide template (110 bases) for the detection of polymerase and exonuclease activities. Polymerase products (65) and exonuclease products (s) are indicated. Protein concentration titration assays (5, 10, 20, 40, 80, 200, 500 and 1000 nM) on DNA- (**A**, **D, E**) or RNA-primed (**C**) templates. Time course assays (2, 4, 7, 10, 15, 20, 30 and 60 minutes) with DNA-primed template and 80 nM PolC (**B**). The reaction samples were resolved by electrophoresis through denaturing 15% (v/v) urea-polyacrylamide gels, the results were analyzed using molecular imager and associated software (Bio-Rad) and the percentage of the assay products was plotted using Graphpad – Prism 6. Bars represent mean values with standard errors of at least three independent protein samples.

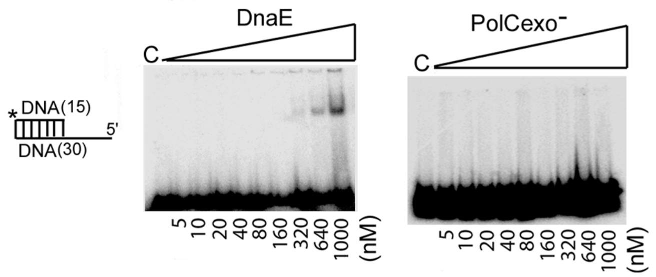

Supplemental Figure S9

Electophoretic Mobility Shift Assays on DnaE and PolC$_{exo-}$.

EMSAs showing the binding affinity of DnaE and PolC$_{exo-}$ to DNA primed (15 nt) templates (30 nt). Protein concentration titration assays were carried out (5, 10, 20, 40, 80, 160, 320, 640 and 1000 nM) in the presence of 0.66 nM radiolabelled template (asterisk) and incubated at 37°C for 5 minutes. Lanes labeled C, represent the control radiolabelled substrate on its own. Reaction samples were resolved by native PAGE and the results were analyzed using molecular imager and associated software (Bio-Rad).

Table S1

Strain	Context	Genotype	Antibiotic resistance	Main phenotypes		Reference or construction[a]	Source
168	168	*trpC2*	-	-		Laboratory collection	
JJS9[b]	168	*trpC-1*	-	-		Laboratory collection	Philippe Noirot
PS1175	168	*trpC2 spoIIAC1*	-	Spo$^-$		(1)	Jeff Errington
PB1856	168	*mutSL::cat trpC2 pheA1*	Cm^R	MutSL$^-$		(2)	Alexandro Galizzi
F25	168	*polC25 polA59 met his leu*	HB^R	PolC mutator		(3)	Neal C. Brown
F27	168	*polC27 polA59 met his leu*	HB^R	PolC mutator		(3)	Neal C. Brown
BD337	168	*mut1 trpC2 thr-5*	-	PolC exo$^-$		(4)	Thomas A. Trautner
L1434	168	*dnaD23 metC lys21*	-	DnaD^{Ts}		(5)	Dimitri Karamata
L1439	168	*dnaI2 metC ilvA1*	-	DnaI^{Ts}		(5)	Dimitri Karamata
EDV97	168	*polA::pmr trpC2*	Phl^R	PolA$^-$		Laboratory collection	Etienne Dervyn
EDJ148	168	*dnaE2.6-pmr trpC2*	Phl^R	DnaE^{Ts}		(6)	Etienne Dervyn
HVS567	168	*recA::tet trpC2*	Tet^R	RecA$^-$		(7)	Etienne Dervyn
HVS597	168	*Pspac-dnaC-ery trpC2*	Em^R	DnaC^{ind}	IPTG dependent	(6)	
HVS609	168	*Pspac-polC-ery trpC2*	Em^R	PolC^{ind}	IPTG dependent	(6)	
HVS614	168	*Pspac-dnaE-ery trpC2*	Em^R	DnaE^{ind}		(6)	
HVS609p	168	*Pspac-polC-ery trpC2* pMAP65	Em^R Km^R	PolC^{ind}	IPTG dependent	Laboratory collection	
HVS614p	168	*Pspac-dnaE-ery trpC2* pMAP65	Em^R Km^R	DnaE^{ind}	IPTG dependent	Laboratory collection	
DGRM1	TF8A	*dnaE2.2-pmr trpC2*	Phl^R	DnaE^{Ts}		(8)	
DGRM2	TF8A	*dnaE2.4-pmr trpC2*	Phl^R	DnaE^{Ts}		(8)	
DGRM3	TF8A	*dnaE2.6-pmr trpC2*	Phl^R	DnaE^{Ts}		(8)	
DGRM4	TF8A	*dnaE2.10-pmr trpC2*	Phl^R	DnaE^{Ts}		(8)	
DGRM630	TF8A	*dnaE2.1-prm trpC2*	Phl^R	DnaE^{Ts}		(8)	
DGRM631	TF8A	*dnaE2.3-prm trpC2*	Phl^R	DnaE^{Ts}		(8)	
DGRM632	TF8A	*dnaE2.5-prm trpC2*	Phl^R	DnaE^{Ts}		(8)	
DGRM633	TF8A	*dnaE2.7-prm trpC2*	Phl^R	DnaE^{Ts}		(8)	
DGRM634	TF8A	*dnaE2.8-prm trpC2*	Phl^R	DnaE^{Ts}		(8)	
DGRM635	TF8A	*dnaE2.11-prm trpC2*	Phl^R	DnaE^{Ts}		(8)	
DGRM799	168	*polC-spc trpC2*	Spc^R			PCR *polC-spc* → 168 (Spc)	
DGRM801	168	*mut1A-spc trpC2*	Spc^R	PolC exo$^-$		DGRM799 → 168 (Pmr)	
DGRM803	168	*dinG::pmr trpC2*	Phl^R	DinG$^-$		PCR *ΔdinG* → 168 (Pmr)	
DGRM804	168	*kapD::spc trpC2*	Spc^R	KapD$^-$		PCR *ΔkapD* → 168 (Spc)	
DGRM806	168	*yprB::spc trpC2*	Spc^R	YprB$^-$		PCR *ΔyprB* → 168 (Spc)	
DGRM808	168	*ppsA::spc trpC2*	Phl^R	PpsA$^-$		PCR *ΔppsA* → 168 (Spc)	
DGRM810	168	*yhaM::spc trpC2*	Spc^R	YhaM$^-$		PCR *ΔyhaM* → 168 (Spc)	
DGRM812	JJS9	*mutSL::cat trpC-1*	Cm^R	MutSL$^-$		PB1856 → JJS9 (Cm)	
DGRM818	168	*Pspac-polC-ery PolA::prm trpC2*	Em^R Phl^R	PolC^{ind} PolA-	IPTG dependent	EDV97 → HVS609 (Pmr)	
DGRM821	168	*dnaE2.6-pmr amyE::Pspank-dnaE-spc- trpC2*	Phl^R Spc^R	DnaE^{Ts} DnaE^{ind}		pDR111-*dnaE* → EDJ148 (Spc)	
DGRM824	168	*dnaE2.6-pmr amyE::Pspank-dnaED1-spc trpC2*	Phl^R Spc^R	DnaE^{Ts} DnaED1^{ind}	IPTG sensitive	pDR111-*dnaED1* → EDJ148 (Spc)	

DGRM825	168	*dnaE2.6-pmr amyE::Pspank-dnaED2-spc trpC2*	Phl^R Spc^R	DnaE^{Ts} DnaED2^{ind}	IPTG sensitive	pDR111-*dnaED2* ➔ EDJ148 (Spc)	
DGRM827	168	*dnaE2.6-pmr amyE::Pspank-dnaED3-spc trpC2*	Phl^R Spc^R	DnaE^{Ts} DnaED3^{ind}	IPTG sensitive	pDR111-*dnaED3* ➔ EDJ148 (Spc)	
DGRM830	168	*dnaE2.6-pmr amyE::Pspank-dnaESPA-spc trpC2*	Phl^R Spc^R	DnaE^{Ts} DnaESPA^{ind}		pFL6 ➔ DGRM821 (Ery)	
DGRM831	168	*dnaE2.6-pmr amyE::Pspank-dnaED1SPA-spc trpC2*	Phl^R Spc^R	DnaE^{Ts} DnaED1SPA^{ind}	IPTG sensitive	pFL6 ➔ DGRM824 (Ery)	
DGRM832	168	*dnaE2.6-pmr amyE::Pspank-dnaED2SPA-spc trpC2*	Phl^R Spc^R	DnaE^{Ts} DnaED2SPA^{ind}	IPTG sensitive	pFL6 ➔ DGRM825 (Ery)	
DGRM833	168	*dnaE2.6-pmr amyE::Pspank-dnaED3SPA-spc trpC2*	Phl^R Spc^R	DnaE^{Ts} DnaED3SPA^{ind}	IPTG sensitive	pFL6 ➔ DGRM827 (Ery)	
DGRM836	168	*dnaE-pmr trpC2*	Phl^R			PCR *dnaE* ➔ EDJ148 (47°C)	
DGRM837	168	*dnaEM7-pmr trpC2*	Phl^R	DnaEM7		PCR *dnaEM7* ➔ EDJ148 (47°C)	
DGRM838	JJS9	*Pspac-dnaEM7-ery trpC-1*	Em^R	DnaEM7^{ind}		HVS614 ➔ DGRM850 (Ery)[c]	
DGRM840	168	*Pspac-polC-ery dnaE-pmr trpC2*	Em^R Phl^R	PolC^{ind}		DGRM836 ➔ HVS609 (Pmr)	
DGRM841	168	*Pspac-polC-ery dnaEM7-pmr trpC2*	Em^R Phl^R	PolC^{ind} DnaEM7		DGRM837 ➔ HVS609 (Pmr)	
DGRM847	168	Pspac-PolC-prm	Phl^R	PolC^{ind}	IPTG dependent	pMutin-Pmr ➔ HVS609 (Pmr)	
DGRM848	JJS9	*Pspac-dnaEM7-ery Pspac-PolC-prm trpC-1*	Em^R Phl^R	PolC^{ind} DnaEM7^{ind}	IPTG dependent	DGRM847 ➔ DGRM838 (Pmr)	
DGRM850	JJS9	*dnaEM7-pmr trpC-1*	Phl^R	DnaEM7		DGRM837 ➔ JJS9 (Pmr)[c]	
DGRM852	JJS9	*dnaE25-pmr trpC-1*	Phl^R	DnaE25		PCR *dnaE25-pmr* ➔ JJS9 (Pmr)	
DGRM853	168	*polA59 polC25-spc met his leu*	Spc^R	PolC25		DGRM799 ➔ F25 (Spc)[c]	
DGRM855	JJS9	*polC25-spc trpC-1*	Spc^R HB^R	PolC25		DGRM853 ➔ JJS9 (HBr)[c]	
DGRM857	JJS9	*polC27 trpC-1*	HB^R	PolC27		F27 ➔ JJS9 (HBr)[c]	
DGRM860	JJS9	*dnaEM7-pmr polC25-spc trpC-1*	Phl^R Spc^R HB^R	DnaEM7 PolC25		DGRM855 ➔ DGRM850 (Spc)	
DGRM861	JJS9	*dnaEM7-pmr polC27 trpC-1*	Phl^R HB^R	DnaEM7 PolC27		DGRM857 ➔ DGRM850 (HB)	
DGRM871	JJS9	*dnaEM7-pmr mutSL::cat trpC-1*	Phl^R Cm^R	DnaEM7 MutSL$^-$		PB1856 ➔ DGRM850 (Cm)	
DGRM872	JJS9	*dnaE25-pmr mutSL::cat trpC-1*	Phl^R Cm^R	DnaE25 MutSL$^-$		PB1856 ➔ DGRM852 (Cm)	
DGRM873	JJS9	*polC25-spc mutSL::cat trpC-1*	Spc^R Cm^R HB^R	PolC25 MutSL$^-$		PB1856 ➔ DGRM855 (Cm)	
DGRM874	JJS9	*polC27 mutSL::cat trpC-1*	HB^R Cm^R	PolC27 MutSL$^-$		PB1856 ➔ DGRM857 (Cm)	
DGRM875	JJS9	*dnaEM7-pmr polC25-spc mutSL::cat trpC-1*	Phl^R Spc^R HB^R Cm^R	DnaEM7 PolC25 MutSL$^-$		PB1856 ➔ DGRM860 (Cm)	
DGRM876	JJS9	*dnaEM7-pmr polC27 mutSL::cat trpC-1*	Phl^R HB^R Cm^R	DnaEM7 PolC27 MutSL$^-$		PB1856 ➔ DGRM861 (Cm)	

Plasmides	main characteristics	antibiotic resistance	Reference or construction[a]	Source
pDR111	Integration vector used to place genes under the control of the IPTG inducible promoter *Phyper-spank* at the *amyE* locus	Spc^R	(9)	Richard Losick
pMUTIN-*prm*	Integration vector used to place genes under the IPTG inducible promoter *Pspac*	Phl^R	Laboratory collection	
pMAP65	Used to over-produce LacI^q	Km^R	(10)	Marie-Agnès Petit
pFL6	pMUTIN derivative designed to insert the SPA tag at the C terminus of *dnaE*	Em^R	(11)	François Lecointe
PDR111-*dnaE*	pDR111 derivative encoding *dnaE* from *Phyper-spank*	Spc^R	PCR *dnaE* ➔ pDR111	
PDR111-*dnaED1*	pDR111 derivative encoding *dnaED1* from *Phyper-spank*	Spc^R	PCR *dnaED1* ➔ pDR112	
PDR111-*dnaED2*	pDR111 derivative encoding *dnaED2* from *Phyper-spank*	Spc^R	PCR *dnaED2* ➔ pDR113	
PDR111-*dnaED3*	pDR111 derivative encoding *dnaED3* from *Phyper-spank*	Spc^R	PCR *dnaED3* ➔ pDR114	

[a] X ➔ Y indicates that strain Y was transformed with DNA from source X, with the selection noted in parentheses: Spc: spectinomycin; Ery: erythromycin; Cat: chloramphenicol ; Pmr: phleomycin; Kan: kanamycin; HB: HB-EMAU.

[b] This strain is a derivative of 168 in which (i) the *trpC2* locus was replaced by a WT allele and (ii) an A was deleted from a run of 6As in the *trpC* gene.

[c] The transfer of the *polC25* and *polC27-spc* mutations in the various strains was selected on HB-EMAU or Spc LB plates. The transfer of the *dnaEM7-pmr, dnaE25-pmr* or *Pspac-dnaE-erm* mutations was carried out using selection on phleomycin or erythromycin. The presence of the polymerase mutation was confirmed by mutagenesis assays and/or DNA sequencing.

References

1. Magill, NG, Setlow, P. 1992 Properties of purified sporlets produced by spoII mutants of Bacillus subtilis. *J. Bact.* **174**, 8148–8151. (doi:10.1128/jb.174.24.8148-8151.1992)

2. Ginetti, F, Perego, M, Albertini, AM, Galizzi, A. 1996 *Bacillus subtilis mutS mutL* operon: identification, nucleotide sequence and mutagenesis. *Microbiology* **142**, 2021–2029. (doi:10.1099/13500872-142-8-2021)

3. Gass, KB, Cozzarelli, NR 1973 Further genetic and enzymological characterization of three *Bacillus subtilis* deoxyribonucleic acid polymerases. *J. Biol. Chem.* **248**, 7688–7700. (http://www.jbc.org/content/248/22/7688.short#ref-list-1)

4. Gross, JD, Karamata, D, Hempstead PG. 1968 Temperature-sensitive mutants of *Bacillus subtilis* affected in DNA synthesis. *Cold Spring Harbor Symp Quant Biol.* **33**, 307. (doi:10.1101/SQB.1968.033.01.034)

5. Karamata, D. & Gross, J. D. 1970 Isolation and genetic analysis of temperature-sensitive mutants of *B. subtilis* defective in DNA synthesis. *Mol Gen Genet* **108**, 277–287. (doi: 10.1007/BF00283358)

6. Dervyn, E., Suski, C., Daniel, R., Bruand, C., Chapuis, J., Errington, J., Jannière, L. & Ehrlich, S. D. 2001 Two essential DNA polymerases at the bacterial replication fork. *Science* **294**, 1716–1719. (doi:http://doi.org/10.1126/science.1066351)

7. Chédin, F., Dervyn, E., Dervyn R., Ehrlich S.D., and Noirot, P. 1994 Frequency of deletion formation decreases exponentially with distance between short direct repeats. *Mol. Microbiol.* **12***,* 561-569. (https://www.ncbi.nlm.nih.gov/pubmed/7934879)

8. Jannière, L. et al. 2007 Genetic evidence for a link between glycolysis and DNA replication. *PLoS ONE* **2**, e447. (doi:10.1371/journal.pone.0000447)

9. van Ooij, C. & Losick, R. 2003 Subcellular localization of a small sporulation protein in *Bacillus subtilis*. *J. Bact.* **185**, 1391–1398. (doi:10.1128/JB.185.4.1391-1398.2003)

10. Petit, M.-A., Dervyn, E., Rose, M., Entian, K.-D., McGovern, S., Ehrlich, S. D. & Bruand, C. 1998 PcrA is an essential DNA helicase of *Bacillus subtilis* fulfilling functions both in repair and rolling-circle replication. *Mol. Microbiol.* **29**, 261–273. (doi: 10.1046/j.1365-2958.1998.00001.x)

11. Costes, A., Lecointe, F., McGovern, S., Quevillon-Cheruel, S. & Polard, P. 2010 The C-Terminal Domain of the Bacterial SSB Protein Acts as a DNA Maintenance Hub at Active Chromosome Replication Forks. *PLoS Genet.* **6**, e1001238. (doi:10.1371/journal.pgen.1001238)
